# Exploration of the Clinical Assessment and Prognostic Value of Society for Cardiovascular Angiography and Intervention Shock Staging in Patients With Acute Myocardial Infarction and Cardiogenic Shock on Veno-arterial Extracorporeal Membrane Oxygenation Support

**DOI:** 10.31083/RCM46066

**Published:** 2026-05-27

**Authors:** Kang Dong, Jing Zhao, Chenliang Pan, Weiting Cai, Juan Ma, Nan Bai, Junchu Wei, Andong Lu, Ming Bai

**Affiliations:** ^1^The First School of Clinical Medicine of Lanzhou University, 730000 Lanzhou, Gansu, China; ^2^Heart Center, The First Hospital of Lanzhou University, 730000 Lanzhou, Gansu, China; ^3^Gansu Province Clinical Research Center for Cardiovascular Diseases, 730000 Lanzhou, Gansu, China

**Keywords:** acute myocardial infarction, cardiogenic shock, extracorporeal membrane oxygenation, enzymes

## Abstract

**Background:**

Although the Society for Cardiovascular Angiography and Intervention (SCAI) five-stage system provides a structured approach to shock assessment, the prognostic utility of this system in patients with acute myocardial infarction and cardiogenic shock (AMICS) supported by venoarterial extracorporeal membrane oxygenation (VA-ECMO), particularly when combined with dynamic cardiac biomarkers, has not been fully validated.

**Methods:**

This single-center, hospital-based, prospective registry study, we continuously enrolled patients with AMICS who received VA-ECMO support at the Cardiac Centre of the First Hospital of Lanzhou University between January 2020 and October 2023, using data from the Chinese Society of Extracorporeal Life Support (CSECLS) registry. Patients were assigned to Stage D or E groups according to the SCAI shock staging system. Clinical characteristics and biomarkers were compared between the two groups, and a multivariate logistic regression model was used to examine the associations among shock stage, cardiac biomarkers, and in-hospital mortality.

**Results:**

This study included 119 patients with AMICS receiving VA-ECMO support, of whom 78 were classified as Stage D and 41 as Stage E. Compared with Stage D patients, Stage E patients had lower VA-ECMO survival scores (–10 vs –2; *p* < 0.001), left ventricular ejection fraction (EF) (0.26 vs 0.33, *p* = 0.050), and high-density lipoprotein cholesterol (HDL-C) levels (0.78 vs 0.89; *p *= 0.013). However, Stage E patients had a higher incidence of ventricular tachycardia and fibrillation (53.7% vs 34.6%, *p *= 0.045), as well as higher glucose levels (14.85 vs 9.54; *p *= 0.016), triglyceride-glucose index (2.29 vs 1.72, *p *= 0.011) and lactate concentration (12.9 vs 9.1; *p *= 0.016). Cardiac troponin I, myoglobin and creatine kinase-MB levels, as well as the associated temporal variation patterns, differed significantly between the two groups. In-hospital mortality rate was significantly higher in Stage E patients than in Stage D individuals (78% vs 53.8%; *p* = 0.010). Multivariate logistic regression analysis showed that combining SCAI shock staging with age, troponin I 6-0, and myoglobin 24-0 significantly improved the prediction of mortality risk (area under the receiver operating characteristic (ROC) curve = 0.791). The 1-year follow-up survival rate was higher in Stage D patients (37.2%) than in Stage E individuals (19.5%).

**Conclusion:**

Combining SCAI shock staging with the dynamic monitoring of cardiac biomarkers facilitates the early risk stratification in patients with AMICS receiving VA-ECMO support, and demonstrates good predictive value for in-hospital mortality.

## 1. Introduction

Cardiogenic shock (CS) is a leading cause of mortality among patients with acute myocardial infarction (AMI) [1]. Global registry studies have reported that acute myocardial infarction and cardiogenic shock (AMICS) occurs in approximately 4–12% of cases and is associated with a 30-day mortality rate of 40–45% [2,3,4,5,6,7]. Despite advances in emergency interventional therapies and novel vasoactive agents, the prognosis of patients with AMICS remains poor [8]. In addition to early revascularisation strategies, short-term mechanical circulatory support such as veno-arterial extracorporeal membrane oxygenation (VA-ECMO) is recommended for patients with AMI presenting with refractory CS. Although various models exist for the prognostic evaluation of critically ill patients, their application in AMICS remains limited.

The Society of Cardiovascular Angiography and Interventions (SCAI) proposed a five-stage classification (A–E) for CS in a clinical expert consensus based on the severity and progression of the condition [9]. These stages are at-risk, beginning, classic, deteriorating, and extreme [10]. This classification offers clinicians a simple and rapid method to assess the severity of CS and guides patient management [10,11,12]. It facilitates initial evaluation and the dynamic monitoring of disease progression and treatment responses, enabling the timely adjustment of therapy [13]. Despite the use of the five-stage classification in risk assessment and the availability of various percutaneous mechanical circulatory support options, AMICS outcomes have changed slightly over the past 30 years. In patients with AMICS receiving VA-ECMO, traditional clinical signs may be obscured, complicating the accurate assessment of recovery or deterioration [10]. Furthermore, some SCAI indicators remain reliant on clinical judgment and are not integrated with dynamic cardiac biomarker evaluation.

We aimed to analyse the baseline clinical characteristics of patients with AMICS on VA-ECMO across different shock stages and investigate the associations among staging, cardiac biomarkers and in-hospital mortality risk to guide clinical management.

## 2. Materials and Methods

### 2.1 Study Population

**Fig. 1.**
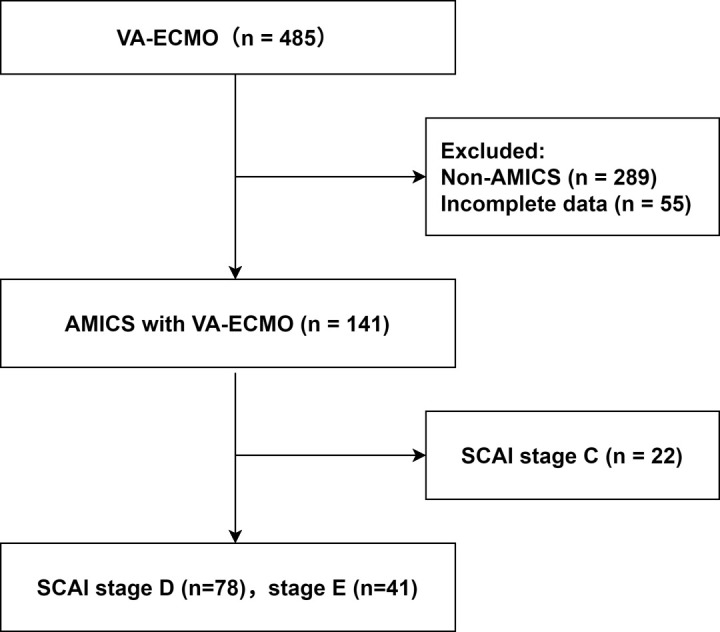
**Flowchart of patient selection based on the inclusion and exclusion criteria**. VA-ECMO, veno-arterial extracorporeal membrane oxygenation; AMICS, acute myocardial infarction complicated by cardiogenic shock; SCAI, Society for Cardiovascular Angiography and Interventions.

This prospective registry study included participants from the Chinese Society of Extracorporeal Life Support (CSECLS) registry database (NCT04158479). We previously published a series of studies based on this registry database [14,15,16,17]. Patients diagnosed with AMICS and treated with VA-ECMO at the Cardiac Centre of Lanzhou University First Hospital between January 2020 and October 2023 were included. In total, 119 patients were selected based on the inclusion and exclusion criteria (Fig. 1). According to the SCAI shock staging criteria, 78 and 41 participants were classified as Stage D and E patients, respectively. This study was approved by the Institutional Review Board of the First Hospital of Lanzhou University, and the requirement for informed consent was waived (Reference: LDYYLL-2025-825).

### 2.2 Inclusion and Exclusion Criteria

The inclusion criteria were age >18 years; the diagnosis of AMICS and treatment with VA-ECMO; and classification consistent with SCAI Stages D and E. The exclusion criteria were incomplete clinical data and the requirement of multiple ECMO treatments.

### 2.3 Definitions and Outcomes

The diagnosis of AMI was established in accordance with the Fourth Universal Definition of Myocardial Infarction [18]. The diagnosis of CS was based on the SCAI CS staging system [10]. In this study, all AMICS patients met the criteria for SCAI CS Stage C or Stage D.

Clinical data were obtained from the CSECLS registry database. The data included general information, medical history, laboratory tests, echocardiography, the Sequential Organ Failure Assessment (SOFA) score , and the Survival After Veno-Arterial ECMO (SAVE) score. Coronary intervention-related results, including infarct-related arteries, pre- and post-procedural thrombolysis in myocardial infarction grades, the number of coronary lesions, the number of stents deployed, and the presence of chronic total occlusion, were obtained using the interventional imaging system of Lanzhou University First Hospital.

Laboratory tests included the measurements of blood glucose (Glu), high-density lipoprotein cholesterol (HDL-C), triglyceride-glucose index (TyG), C-reactive protein (CRP), interleukin-6 (IL-6), lymphocyte percentage (LYM%), monocyte percentage (MONO%), eosinophil percentage (EO%), lactate (Lac), serum potassium (K^+^) and sodium (Na^+^), as well as cardiac enzymes measured at admission and at 6 and 24 h post-operatively. The evaluated cardiac enzymes included cardiac troponin I (TnI), creatine kinase-MB (CK-MB), myoglobin (Myo), N-terminal pro-B-type natriuretic peptide (NT-proBNP), and D-dimer. Biomarker levels were recorded at three pre-defined time points: 0 h (on admission), 6 h (6 h after percutaneous coronary intervention [PCI]), and 24 h (24 h after PCI). Δ(6 - 0 h) represented the difference between the biomarker levels at 6 h post-PCI and at admission; Δ(24 - 0 h) indicated the difference between the biomarker levels at 24 h post-PCI and at admission; Δ(24 - 6 h) denoted the difference between the biomarker levels at 24 h and 6 h post-PCI.

Echocardiographic assessments included the measurements of left ventricular ejection fraction (EF) and fractional shortening (FS), as well as the evaluation of structural abnormalities such as ventricular aneurysm, myocardial perforation, and chordae tendineae rupture.

Coronary angiography included the assessment of the left main coronary artery, left anterior descending artery (LAD), left circumflex artery, and right coronary artery.

### 2.4 Statistical Analysis

All data were processed and statistically analysed using IBM SPSS Statistics (version 26.0; IBM Corporation, Armonk, NY, USA). Quantitative variables following a normal or approximately normal distribution were expressed as mean ± standard deviation, and group comparisons were conducted using the *t*-test. For non-normally distributed quantitative variables, data were presented as median and interquartile range (M [Q1–Q3]), with group comparisons performed using the Wilcoxon Mann–Whitney test. Categorical variables were reported as numbers and/or percentages (N [%]), and group comparisons were conducted using the chi-square test, continuity correction chi-square test, or Fisher’s exact test. To explore the independent associations of SCAI staging and biological markers with in-hospital mortality, a multivariable logistic regression model was constructed. Collinearity diagnostics, including variance inflation factor (VIF), were performed during model development to ensure model stability. Model discrimination was assessed using receiver operating characteristic (ROC) curves and the area under the curve (AUC), whereas calibration was evaluated using the Hosmer–Lemeshow goodness-of-fit test or calibration plots. Internal validation was performed using 1000 bootstrap resamples to assess the model’s calibration and discriminatory performance. Furthermore, 1-year survival rates were analysed using Kaplan–Meier survival curves, and statistical differences were evaluated using the log-rank test. A two-tailed test was used, and a *p*-value < 0.05 was considered statistically significant.

## 3. Results

### 3.1 Comparison of Clinical Baseline Characteristics

Among the 119 study participants with AMICS on VA-ECMO support, 18 (15.1%) were women. The median age of all patients was 62 (54–70) years. The timing of AMI onset differed significantly between the two groups. The incidences of ventricular tachycardia and ventricular fibrillation were considerably higher in the E-stage group. In the E-stage group, the median total hospital stay and cardiac care unit admission time were 4 days, considerably shorter than those in the D-stage group. The median SAVE score in the E-stage group was –10, significantly lower than that in the D-stage group. The mortality rate was 53.8% in the D-stage group, compared with 78% in the E-stage group. All differences were statistically significant (*p *< 0.05). The baseline characteristics are summarised in Table 1.

**Table 1.** **Baseline characteristics and clinical profile of patients**.

			Total (n = 119)	Stage E (n = 41)	Stage D (n = 78)	*p*
Demographics						
	Age, median (25th and 75th), years		62 (54, 70)	68 (54, 72)	61 (54, 69)	0.329
	Female, n (%)		18 (15.1%)	3 (7.3%)	15 (19.2%)	0.085
	BMI, median (25th and 75th), kg/m^2^		23.9 (22.0, 25.7)	24.2 (22.1, 25.7)	23.7 (21.8, 25.8)	0.778
	Smoking, n (%)		90 (75.6%)	33 (80.5%)	57 (73.1%)	0.371
Medical history						
	Hypertension, n (%)		60 (50.4%)	22 (53.7%)	38 (48.7%)	0.608
	Hyperlipidaemia, n (%)		4 (3.4%)	2 (4.9%)	2 (2.6%)	0.896
	Diabetes mellitus, n (%)		39 (32.8%)	14 (34.1%)	25 (32.1%)	0.817
	Chronic kidney disease, n (%)		1 (0.8%)	1 (2.4%)	0 (0%)	0.345
	Coronary artery disease, n (%)		33 (27.7%)	11 (26.8%)	22 (28.2%)	0.873
	PCI, n (%)		29 (24.4%)	11 (26.8%)	18 (23.1%)	0.650
	Atrial fibrillation, n (%)		2 (1.7%)	2 (4.9%)	0 (0%)	0.117
	Acute heart failure, n (%)		2 (1.7%)	0 (0%)	2 (2.6%)	0.545
Admission assessment findings						
	Heart rate, median (25th and 75th), beats/minute		92.5 (71.8, 108.3)	90.5 (70.1, 108.3)	92.5 (72.8, 108.3)	0.941
	Ventricular tachycardia/fibrillation, n (%)		49 (41.2%)	22 (53.7%)	27 (34.6%)	0.045
	SOFA score, median (25th and 75th)		10 (8, 12)	11 (8, 12)	9 (8, 11.25)	0.079
	SAVE score, median (25th and 75th)		–5 (–10, –1)	–10 (–11.5, –8)	–2 (–7, –1)	<0.001
Onset time of AMI						
	<12 h, n (%)		55 (46.2%)	26 (63.4%)	29 (37.2%)	0.021
	12, 24 h, n (%)		21 (17.6%)	4 (9.8%)	17 (21.8%)	
	>24 h, n (%)		43 (36.1%)	11 (26.8%)	32 (41.0%)	
CAG (stenosis >50%)						
	LM, n (%)		19 (16.0%)	7 (17.1%)	12 (15.4%)	0.811
	LAD, n (%)		86 (72.3%)	31 (75.6%)	55 (70.5%)	0.555
	LCX, n (%)		73 (61.3%)	30 (73.2%)	43 (55.1%)	0.055
	RCA, n (%)		77 (64.7%)	29 (70.7%)	48 (61.5%)	0.319
Occluded artery						
	LM, n (%)		8 (6.7%)	2 (4.9%)	6 (7.7%)	0.843
	LAD, n (%)		39 (32.8%)	11 (26.8%)	28 (35.9%)	0.317
	LCX, n (%)		19 (16.0%)	6 (14.6%)	13 (16.7%)	0.774
	RCA, n (%)		40 (33.6%)	16 (39.0%)	24 (30.8%)	0.365
	≥2-vessel coronary artery disease, n (%)		87 (73.1%)	30 (73.2%)	57 (73.1%)	0.991
	Presence of ≥1 coronary artery CTO, n (%)		83 (69.7%)	29 (70.7%)	54 (69.2%)	0.865
	Number of stents implanted, median (25th and 75th)		1 (1, 2)	1 (1, 2.5)	2 (1, 2.25)	0.457
	Pre-procedural TIMI flow grade 0, n (%)		85 (71.4%)	29 (70.7%)	56 (71.8%)	0.903
Echocardiography						
	EF, median (25th and 75th), %		0.3 (0.22, 0.41)	0.26 (0.19, 0.32)	0.33 (0.23, 0.42)	0.050
	FS, median (25th and 75th), %		15 (11, 20)	12.5 (10, 16)	17 (12, 21)	0.009
	Ventricular aneurysm, n (%)		12 (10.1%)	4 (9.8%)	8 (10.3%)	>0.999
	Perforation, n (%)		4 (3.4%)	1 (2.4%)	3 (3.8%)	>0.999
	Chordae tendineae rupture, n (%)		5 (4.2%)	0 (0%)	5 (6.4%)	0.240
Laboratory test						
	Glu, median (25th and 75th), mmol/L		11.06 (7.33–17.68)	14.85 (8.67, 21.31)	9.54 (6.86, 16.72)	0.016
	HDL-C, median (25th and 75th), mmol/L		0.81 (0.60, 1.04)	0.78 (0.54, 0.86)	0.89 (0.69, 1.08)	0.013
	TyG, median (25th and 75th), mg/dL		1.89 (1.44, 2.53)	2.29 (1.64, 3.02)	1.72 (1.37, 2.44)	0.011
	CRP, median (25th and 75th), mg/L		12.2 (3.9, 64.4)	5.0 (1.8, 22.2)	26.37 (7.1, 84.7)	<0.001
	IL-6, median (25th and 75th), pg/mL		137.5 (73.5, 375.8)	321 (125.8, 2112.5)	108.5 (64.0, 194.3)	0.001
	LYM%, median (25th and 75th)		8.3 (6, 17.4)	10.5 (6.45, 22.8)	7.55 (5.38, 13.85)	0.028
	MONO%, median (25th and 75th)		3.9 (2.6, 5.8)	2.8 (2.4, 5.6)	4.45 (3, 5.9)	0.016
	EO%, median (25th and 75th)		0.1 (0, 0.4)	0.2 (0, 0.85)	0 (0, 0.1)	<0.001
	Lac, median (25th and 75th), mmol/L		10 (7.25, 14.15)	12.9 (8.1, 15.20)	9.2 (6.1, 13.4)	0.016
	K^+^, median (25th and 75th), mmol/L		4.1 (3.6, 4.8)	3.8 (3.25, 4.68)	4.15 (3.79, 4.81)	0.013
	Na^+^, median (25th and 75th), mmol/L		140.4 (137, 144)	142 (137.55, 146)	140 (136.68, 142.3)	0.010
Outcomes						
	ECMO-related complications					
		Intracranial haemorrhage, n (%)	3 (2.5%)	1 (2.4%)	2 (2.6%)	>0.999
		Gastrointestinal bleeding, n (%)	13 (10.9%)	5 (12.2%)	8 (10.3%)	0.990
		AKI, n (%)	52 (43.7%)	16 (39%)	36 (46.2%)	0.456
		Duration of ECMO support, median (25th and 75th), h	102.0 (47.0, 152.5)	58.3 (28.0, 107.3)	117.5 (72.5, 176.5)	<0.001
		Total hospital stay, median (25th and 75th), days	8 (4, 17)	4 (1, 12)	10.5 (4.75, 18.25)	0.002
		ICU stay, median (25th and 75th), days	7 (4, 14)	4 (1, 10.5)	9 (4, 16.25)	0.001
		In-hospital mortality rate, n (%)	74 (62.2%)	32 (78%)	42 (53.8%)	0.010

BMI, body mass index; PCI, percutaneous coronary intervention; SOFA, Sequential Organ Failure Assessment; SAVE, Survival After Veno-arterial ECMO; AMI, acute myocardial infarction; CAG, coronary angiography; LM, left main coronary artery; LAD, left anterior descending artery; LCX, Left circumflex artery; RCA, right coronary artery; CTO, chronic total occlusion; TIMI, thrombolysis in myocardial infarction; EF, ejection fraction; FS, fractional shortening; Glu, blood glucose; HDL-C, high-density lipoprotein cholesterol; TyG, triglyceride-glucose index; CRP, C-reactive protein; IL-6, interleukin-6; LYM%, lymphocyte percentage; MONO%, monocyte percentage; EO%, eosinophil percentage; Lac, lactate; K^+^, serum potassium; Na^+^, serum sodium; ECMO, extracorporeal membrane oxygenation; AKI, acute kidney injury; ICU, intensive care unit.

### 3.2 Coronary Intervention-Related Results Between the Two Groups

All study participants underwent PCI. The LAD had the highest proportion of lesions, and >50% stenosis was observed in 86 patients (72.3%). Forty patients (33.6%) had RCA occlusion. Eighty-five patients (71.4%) exhibited a pre-procedural thrombolysis in myocardial infarction (TIMI) flow grade of 0. The median number of implanted stents was 1 (1, 2). No statistically significant differences were found between the two groups for the above parameters (all *p* > 0.05). The results comparing coronary intervention between the two groups are presented in Table 1.

### 3.3 Echocardiographic and Laboratory Findings Between the Two Groups

Echocardiographic examination revealed that the median EF and FS in the E-stage group were 0.26 and 12.5, respectively, which were significantly lower than those in the D-stage group (*p *< 0.05). The median Glu, TyG, and IL-6 levels were significantly higher in the E-stage group than in the D-stage group (*p *< 0.05). The median levels of HDL-C and CRP were significantly lower in the E-stage group than in the D-stage group (*p *< 0.05). Among the blood gas analysis parameters, Lac, potassium ion, and sodium ion concentrations differed significantly between the two groups (*p *< 0.05). However, the other parameters showed no significant differences between the two groups (*p *> 0.05). The echocardiographic and laboratory findings of the two groups are presented in Table 1. Detailed laboratory parameters are summarised in **Supplementary Table 1**. In addition, lactate levels were evaluated using both static and dynamic analyses. In the static analysis, significant differences between Stage D and Stage E were observed at 0 h, 6 h, and 24 h. In contrast, no statistically significant differences were identified in the dynamic analyses of Δ(6 - 0 h), Δ(24 - 0 h), or Δ(24 - 6 h). The detailed results of lactate-related analyses are presented in **Supplementary Table 2**.

### 3.4 Myocardial Enzyme-Related Results Between the Two Groups

**Fig. 2.**
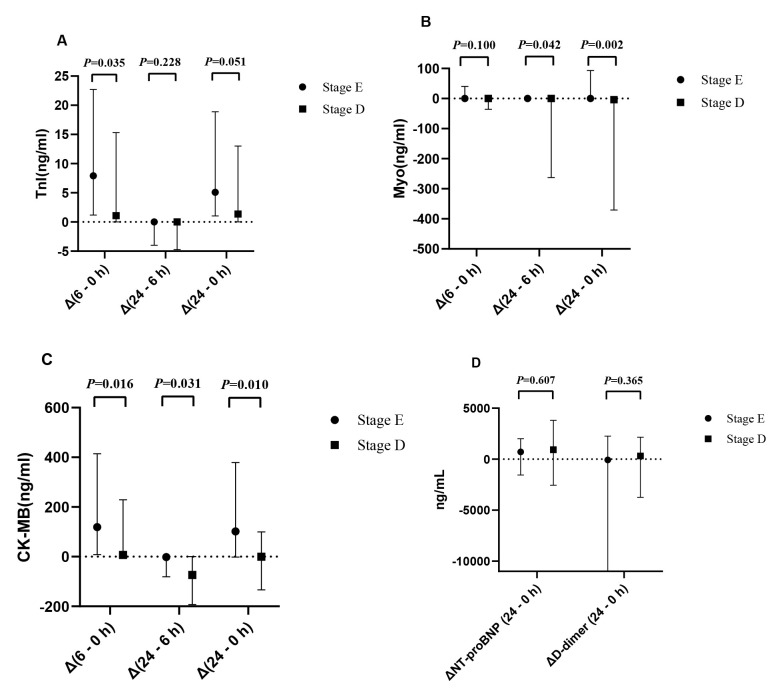
**Time-based analysis of myocardial enzyme changes in patients with AMICS**. (A) Dynamic analysis of TnI. (B) Dynamic analysis of Myo. (C) Dynamic analysis of CK-MB. (D) Dynamic analysis of NT-proBNP and D-dimer. Δ(6 - 0 h): Difference between the biomarker levels at 6 h post-PCI and at admission; Δ(24 - 6 h): Difference between the biomarker levels at 24 and 6 h post-PCI; Δ(24 - 0 h): Difference between the 24 h post-PCI levels and those at admission.

In both groups, myocardial enzyme levels increased initially and subsequently declined. The first myocardial enzyme tests after admission (Table 2) showed that the median TnI levels differed significantly between the two groups, measuring 1.5 and 8 ng/mL in the E- and D-stage groups, respectively (*p *< 0.05). Additionally, the two groups exhibited significant differences in NT-proBNP, D-dimer, and Myo levels at admission; however, only their Myo levels differed significantly at 6 and 24 h after PCI (*p *< 0.05). The changes in myocardial enzyme levels at admission (Fig. 2), as well as at 6 and 24 h after PCI, were analysed. “Above the zero baseline” indicates an increase in the indicator, and “below the zero baseline” indicates a decrease. The D- and E-stage groups showed significant increases in TnI levels at 6 h after surgery compared with those at admission; the increase was more pronounced in the E-stage group. In the E-stage group, Myo levels at 24 h post-surgery were less than those at 6 h post-surgery and at admission. CK-MB levels showed no significant decrease among the three time points: at admission, 6 h post-surgery, and 24 h post-surgery. These indicators differed significantly between the two groups (*p *< 0.05).

**Table 2.** **Myocardial enzyme-related results between the two groups**.

		All patients (n = 119)	Stage E (n = 41)	Stage D (n = 78)	*p*
Hospital admission					
	TnI, median (25th and 75th), ng/mL	4.6 (0.8, 23)	1.5 (0.5, 7.3)	8 (1.2, 25)	0.003
	CK-MB, median (25th and 75th), ng/mL	85 (25, 400)	53.5 (17.3, 246.8)	104 (30.5, 473.5)	0.076
	Myo, median (25th and 75th), ng/mL	900 (666, 900)	900 (771.5, 900)	900 (593, 900)	0.553
	NT-proBNP, median (25th and 75th), pg/mL	3720 (758, 9420)	2145 (397.5, 5597.5)	5330 (1580, 10,800)	0.009
	D-dimer, median (25th and 75th), ng/mL	3220 (856.3, 13,450)	10,515 (1064.5, 35,050)	2500 (852.8, 7310)	0.039
6 h postoperatively					
	TnI, median (25th and 75th), ng/mL	25 (7, 25)	22 (3.3, 25)	25 (8.6, 25)	0.198
	CK-MB, median (25th and 75th), ng/mL	431 (71, 500)	500 (96, 500)	412 (47, 500)	0.554
	Myo, median (25th and 75th), ng/mL	900 (614, 900)	900 (900, 900)	900 (530, 900)	0.033
24 h postoperatively					
	TnI, median (25th and 75th), ng/mL	14 (5.2, 25)	11 (3.1, 25)	18 (6.5, 25)	0.172
	CK-MB, median (25th and 75th), ng/mL	200 (50.8, 432.8)	258 (78, 500)	179 (47, 396)	0.259
	Myo, median (25th and 75th), ng/mL	900 (474, 900)	900 (900, 900)	823.5 (302.3, 900)	0.001

TnI, troponin I; CK-MB, creatine kinase-MB isoenzyme; Myo, myoglobin; NT-proBNP, N-terminal pro-brain natriuretic peptide.

### 3.5 Factors Influencing In-Hospital Mortality in Patients With AMICS on VA-ECMO Support

According to the multivariate logistic regression analysis, age, SCAI shock staging, ΔTnI (6 - 0 h) levels, and ΔMyo (24 - 0 h) levels were statistically significant predictors of mortality in patients on VA-ECMO support. The analysis showed that SCAI shock staging (odds ratio [OR] = 0.350, 95% confidence interval [CI]: 0.133–0.917) combined with age (OR = 1.052, 95% CI: 1.013–1.093), ΔTnI (6 - 0 h) levels (OR = 0.954, 95% CI: 0.911–0.998) and ΔMyo (24 - 0 h) (OR = 1.002, 95% CI: 1.000–1.004) levels were independently associated with in-hospital mortality in patients with AMICS on VA-ECMO support. A risk prediction model for in-hospital mortality in patients with AMICS on VA-ECMO support was developed based on these four factors. The factors affecting in-hospital mortality in patients with AMICS on VA-ECMO are presented in Table 3. The model’s performance showed no substantive change after adjusting for confounding factors (**Supplementary Table 3**).

**Table 3.** **Multivariable logistic regression analysis for in-hospital mortality in patients with AMICS receiving VA-ECMO support**.

	B	*p*	OR	95% CI
Age	0.051	0.009	1.052	1.013–1.093
SCAI Shock Staging (Stage E)	–1.051	0.033	0.350	0.133–0.917
ΔTnI (6 - 0 h)	–0.047	0.043	0.954	0.911–0.998
ΔMyo (24 - 0 h)	0.002	0.014	1.002	1.000–1.004

SCAI, Society for Cardiovascular Angiography and Interventions; ΔTnI (6 - 0 h), the difference between the troponin I levels at 6 h post-PCI and at admission; ΔMyo (24 - 0 h), the difference between the myoglobin levels at 24 h post-PCI and at admission; OR, odds ratio; 95% CI, confidence interval.

### 3.6 Predictive Value of SCAI Shock Staging for In-Hospital Mortality in Patients With AMICS on VA-ECMO Support

**Fig. 3.**
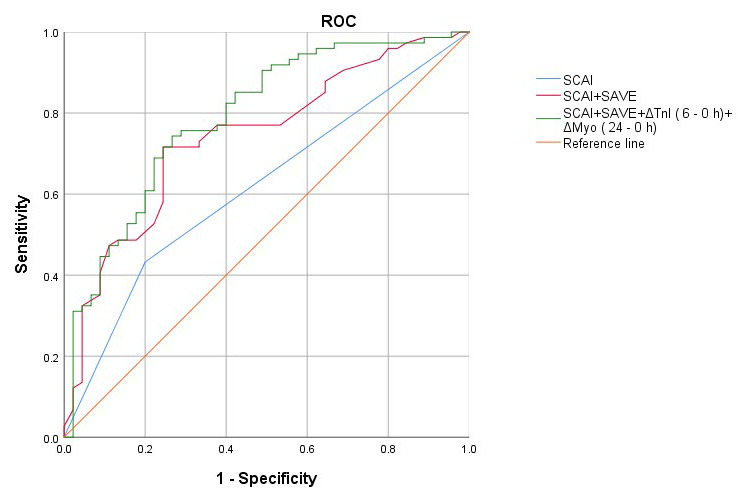
**Receiver operating characteristic (ROC) curves of the prediction model and other indicators predicting outcome in patients receiving VA-ECMO**.

The AUC for predicting in-hospital mortality using SCAI shock staging, a predictive model combining SCAI shock staging and the SAVE score, and a predictive model combining SCAI shock staging, age, ΔTnI (6 - 0 h) levels, and ΔMyo (24 - 0 h) levels were 0.616, 0.741, and 0.791, respectively. Combined SCAI shock staging, age, ΔTnI (6 - 0 h) levels, and ΔMyo (24 - 0 h) levels enhanced the prediction of mortality risk. The model showed no evidence of multicollinearity (**Supplementary Tables 4,5,6**), demonstrated good calibration (**Supplementary Table 7**), and exhibited effective internal validation (**Supplementary Table 8**). Temporal internal validation was also performed (**Supplementary Figs. 1,2**). The predictive value of the SCAI shock staging for in-hospital mortality in patients with AMICS on VA-ECMO is shown in Fig. 3.

### 3.7 Survival Outcomes of Patients With Different SCAI Shock Stages

**Fig. 4.**
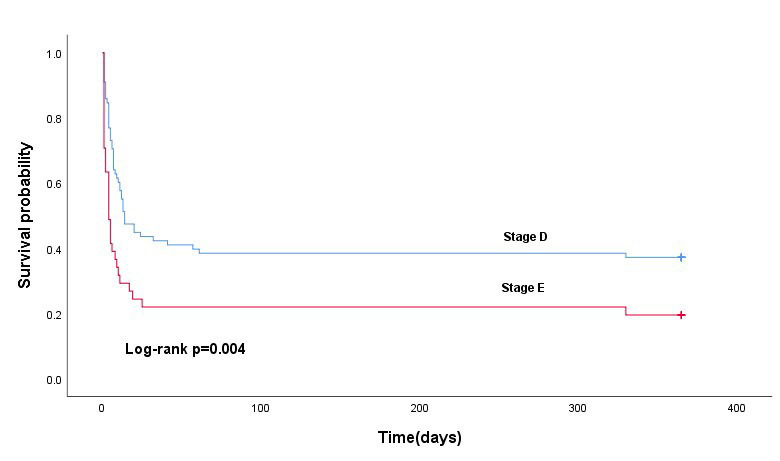
**Kaplan–Meier survival curves depicting 1-year survival rates for different SCAI shock stages in patients with AMICS treated with VA-ECMO**.

The overall in-hospital survival rate of patients with AMICS included in this study was 37.8%, and the 1-year survival rate was 31.1%. Among in-hospital survivors, 9 (22.0%) and 36 (46.2%) were in the E- and D-stage groups, respectively. The Kaplan–Meier survival curve showed that the 1-year survival rate was higher in the D-stage group (29 patients [37.2%]) than in the E-stage group (eight patients [19.5%]) (*p *= 0.004). The survival outcomes of patients with different SCAI shock stages are shown in Fig. 4.

## 4. Discussion

We examined the clinical characteristics of patients with AMICS on VA-ECMO support across different SCAI shock stages. We found that a model incorporating SCAI stage, age, ΔTnI (6 - 0 h) levels, and ΔMyo (24 - 0 h) levels achieved an AUC of 0.791, suggesting that the dynamic trajectories of cardiac enzymes are crucial for evaluating disease severity and prognostic risk in AMICS.

The mortality rates observed at the E and D stages in this study were consistent with those reported in international cohorts. The single-centre data of this study showed that the in-hospital mortality rate of patients with AMICS receiving VA-ECMO was 62.2%, and the mortality rate significantly increased with increasing SCAI shock stage. The mortality rate among Stage E patients was 78%, whereas that among Stage D patients was 53.8%. Several studies have shown that the mortality rate of Stage D patients ranges from 24% to 25.2%, whereas that of Stage E patients ranges from 37.7% to 71.4%. [10,19,20]. These findings are consistent with those of the present study.

The analysis of the time from AMI onset to ECMO support revealed that 63.4% of Stage E patients received ECMO support within 12 h, compared with 37.2% of Stage D patients. This difference may be related to the patient’s perception of the urgency of their condition. The American College of Cardiology study and other reports indicate that Stage E patients exhibit more severe symptoms that align more closely with their expectations of a heart attack, prompting them to seek medical attention earlier [9]. Fu *et al.* [21] demonstrated that for patients with AMI, every 30-min delay in reperfusion therapy increases in-hospital mortality by 7.5%. Stage E patients are more likely to experience pre-hospital mortality after 12 h of onset than Stage D patients, which may explain the observed lower proportion of Stage E patients receiving treatment within 12–24 h after onset.

With respect to biochemical and inflammatory markers, the findings of this study were consistent with those reported previously. Stage E patients had lower HDL-C and CRP levels than did Stage D patients; however, Glu, TyG, IL-6, and Lac levels were higher in Stage E individuals. Previous studies have shown that elevated TyG levels are closely associated with poor outcomes in various cardiovascular diseases [22,23]. A Middle East study reported that low HDL-C levels were significantly associated with male in-hospital mortality and the incidence of CS [24]. Systemic inflammatory responses and neurohormonal activation play key roles in the pathophysiology of CS [25,26,27]. In CS, various inflammatory markers and cytokines such as IL-1, IL-6, IL-8, and tumour necrosis factor-alpha are elevated [28]. CRP typically rises progressively and peaks between 48 and 72 h of onset [29], which may account for the lower CRP levels observed in Stage E patients in this cohort. Stage E patients also exhibited significantly higher lactate levels at admission, reflecting more severe tissue hypoperfusion. However, in the multivariable logistic regression analysis, these markers combined with SCAI staging did not yield an optimal model fit.

This study focused on the impact of myocardial enzyme levels at admission and post-operatively, as well as their dynamic changes relative to the shock stage. Previous studies have demonstrated that the extent of elevation of specific cardiac biomarkers is a key prognostic indicator of long-term outcomes in patients undergoing left main PCI. For example, Wang *et al.* [30] emphasised the prognostic significance of post-procedural cardiac biomarker elevation after left main PCI. Regarding acute fulminant myocarditis and CS supported by ECMO, Chou *et al.* [31] identified peak CK-MB levels as a critical prognostic factor for cardiac function recovery. Similarly, Chong *et al.* [32] observed that non-survivors exhibited significantly higher peak levels of TnI than did survivors. In addition, Jarai *et al.* [33] demonstrated that NT-proBNP levels could predict 30-day survival in patients with CS, particularly among those who achieved successful revascularisation. These findings suggest that peri-interventional or peri-support biomarker dynamics provide valuable prognostic information. However, there is limited evidence regarding the combined use of dynamic cardiac biomarker changes and clinical staging systems such as the SCAI shock classification in predicting outcomes among high-risk populations, particularly in patients with AMICS on VA-ECMO. Therefore, we analysed the dynamic changes in myocardial enzyme levels between Stage D and E patient groups.

In the present study, we assessed SCAI shock staging based on patients’ admission status and found that the condition of Stage D patients may deteriorate after admission, as reflected by changes in TnI, CK-MB, and Myo levels from admission to post-surgery. Furthermore, multivariate logistic regression analysis indicated age, SCAI shock staging, ΔTnI (6 - 0 h) levels, and ΔMyo (24 - 0 h) levels as statistically significant predictors of mortality in patients on VA-ECMO support.

In this study, the AUC for predicting in-hospital mortality using SCAI was 0.616, whereas combining SCAI shock staging with the SAVE score increased the AUC to 0.741. The SAVE score alone did not provide strong predictive value for shock staging in this cohort. In patients with AMICS on VA-ECMO support, the combination of SCAI shock staging, age, ΔTnI (6 - 0 h) levels, and ΔMyo (24 - 0 h) levels increased the AUC for predicting in-hospital mortality to 0.791. Compared with the efficiency of SCAI shock staging alone or its combination with the SAVE score, the inclusion of age, ΔTnI (6 - 0 h) levels, and ΔMyo (24 - 0 h) levels alongside SCAI shock staging enhanced the prediction of mortality risk. Further multicentre clinical studies are required to externally validate these results.

In further subgroup and dynamic biomarker trajectory analyses (E-stage results in **Supplementary Tables 9,10,11**; D-stage results in **Supplementary Tables 12,13,14**), ΔCK-MB (24 - 6 h), ΔMyo (24 - 6 h), and ΔMyo (24 - 0 h) showed significant differences between survivors and non-survivors in univariable analyses during the D stage; however, these associations were not retained in the multivariable model (**Supplementary Table 15**).

## 5. Limitations

This study had certain limitations. First, this study was conducted at a single centre with a relatively small sample size (41 cases in the SCAI Stage E group), which may affect the statistical power and generalisability of the results. Hence, multicentre studies with larger samples and external validation are required. Second, the database lacked long-term follow-up information. Finally, although CS is the primary indication for VA-ECMO, it has various aetiologies, including AMI, fulminant myocarditis, advanced cardiomyopathy, advanced valvular disease, and severe arrhythmias. This study focused solely on CS caused by AMI.

## 6. Conclusions

This study validated the clinical utility of the SCAI shock staging in patients with AMICS on VA-ECMO support. Additionally, dynamic monitoring of cardiac enzymes plays a crucial role in assessing the severity and prognostic risk of AMICS. Furthermore, combining SCAI shock staging with age, ΔTnI (6 - 0 h) levels, and ΔMyo (24 - 0 h) levels may enhance the prediction of mortality risk.

## Data Availability

Due to ethical and legal restrictions, individual patient data will not be publicly available but can be accessed upon reasonable request and with appropriate institutional approvals.
