# Supplementary material for: Exploration of the Clinical Assessment and Prognostic Value of Society for Cardiovascular Angiography and Intervention Shock Staging in Patients With Acute Myocardial Infarction and Cardiogenic Shock on Veno-arterial Extracorporeal Membrane Oxygenation Support
Source: Rev Cardiovasc Med. 2026 May 27;27(5):46066. doi: 10.31083/RCM46066 (PMC13227360; doi:10.31083/RCM46066)
Supplement: Supplementary file 1 [file 2153-8174-27-5-46066-s1.zip › Supplementary Material.docx]

**Supplementary Table 1.** Baseline characteristics and clinical profile of patients.

|  | **Total (n=119)** | **Stage E (n=41)** | **Stage D (n=78)** | ***P*** |
| --- | --- | --- | --- | --- |
| **Demographic** |  |  |  |  |
| Age, median (25th and 75th), years | 62 (54, 70) | 68 (54, 72) | 60.5 (54, 69.25) | 0.329 |
| Female, n (%) | 18 (15.1%) | 3 (7.3%) | 15 (19.2%) | 0.085 |
| BMI, median (25th and 75th), kg/m^2^ | 23.88 (22.04, 25.71) | 24.17 (22.07, 25.74) | 23.73 (21.84, 25.77) | 0.778 |
| Smoking, n (%) | 90 (75.6%) | 33 (80.5%) | 57 (73.1%) | 0.371 |
| **Medical history** |  |  |  |  |
| Hypertension, n (%) | 60 (50.4%) | 22 (53.7%) | 38 (48.7%) | 0.608 |
| Hyperlipidemia, n (%) | 4 (3.4%) | 2 (4.9%) | 2 (2.6%) | 0.896 |
| Diabetes mellitus, n (%) | 39 (32.8%) | 14 (34.1%) | 25 (32.1%) | 0.817 |
| Chronic kidney disease, n (%) | 1 (0.8%) | 1 (2.4%) | 0 (0%) | 0.345 |
| Coronary artery disease, n (%) | 33 (27.7%) | 11 (26.8%) | 22 (28.2%) | 0.873 |
| Prior PCI, n (%) | 29 (24.4%) | 11 (26.8%) | 18 (23.1%) | 0.650 |
| Atrial fibrillation, n (%) | 2 (1.7%) | 2 (4.9%) | 0 (0%) | 0.117 |
| Acute heart failure, n (%) | 2 (1.7%) | 0 (0%) | 2 (2.6%) | 0.545 |
| **Admission assessment findings** |  |  |  |  |
| Heart rate, median (25th and 75th), beats/minute | 92.5 (71.75, 108.25) | 90.5 (70.25, 108.25) | 92.5 (72.75, 108.25) | 0.941 |
| Systolic blood pressure, median (25th and 75th), mmHg | 99.5 (83.25, 117) | 89.5 (73, 109.25) | 105 (86, 120.75) | 0.011 |
| Diastolic blood pressure, median (25th and 75th), mmHg | 64.5 (50, 75) | 60 (46, 73) | 67 (54, 79.5) | 0.041 |
| Mean arterial pressure, median (25th and 75th), mmHg | 76.17 (62.08, 88.67) | 68.83 (59.08, 86) | 79.17 (68.67, 93.25) | 0.016 |
| Ventricular tachycardia/fibrillation, n (%) | 49 (41.2%) | 22 (53.7%) | 27 (34.6%) | 0.045 |
| SOFA score, median (25th and 75th) | 10 (8, 12) | 11 (8, 12) | 9 (8, 11.25) | 0.079 |
| SAVE score, median (25th and 75th) | -5 (-10, -1) | -10 (-11.5, -8) | -2 (-7, 1) | <0.001 |
| **Treatment after admission** |  |  |  |  |
| Continuous renal replacement therapy, n (%) | 61 (51.3%) | 24 (58.5%) | 37 (47.4%) | 0.250 |
| Invasive mechanical ventilation, n (%) | 84 (70.6%) | 38 (92.7%) | 46 (59.0%) | <0.001 |
| Non-invasive mechanical ventilation, n (%) | 28 (23.5%) | 8 (19.5%) | 20 (25.6%) | 0.454 |
| Intra-aortic balloon pump support, n (%) | 65 (54.6%) | 21 (51.2%) | 44 (56.4%) | 0.589 |
| Temporary pacemaker support, n (%) | 19 (16.0%) | 5 (12.2%) | 14 (17.9%) | 0.415 |
| PCI, n (%) | 107 (89.9%) | 37 (90.2%) | 70 (89.7%) | >0.999 |
| **Onset time of AMI** |  |  |  |  |
| <12 h, n (%) | 55 (46.2%) | 26 (63.4%) | 29 (37.2%) | 0.021 |
| 12–24 h, n (%) | 21 (17.6%) | 4 (9.8%) | 17 (21.8%) |  |
| >24 h, n (%) | 43 (36.1%) | 11 (26.8%) | 32 (41.0%) |  |
| **CAG (stenosis > 50%)** |  |  |  |  |
| **LM**, n (%) | 19 (16.0%) | 7 (17.1%) | 12 (15.4%) | 0.811 |
| LAD, n (%) | 86 (72.3%) | 31 (75.6%) | 55 (70.5%) | 0.555 |
| LCX, n (%) | 73 (61.3%) | 30 (73.2%) | 43 (55.1%) | 0.055 |
| RCA, n (%) | 77 (64.7%) | 29 (70.7%) | 48 (61.5%) | 0.319 |
| **Occluded artery** |  |  |  |  |
| **LM**, n (%) | 8 (6.7%) | 2 (4.9%) | 6 (7.7%) | 0.843 |
| LAD, n (%) | 39 (32.8%) | 11 (26.8%) | 28 (35.9%) | 0.317 |
| LCX, n (%) | 19 (16.0%) | 6 (14.6%) | 13 (16.7%) | 0.774 |
| RCA, n (%) | 40 (33.6%) | 16 (39.0%) | 24 (30.8%) | 0.365 |
| ≥2-vessel coronary artery disease, n (%) | 87 (73.1%) | 30 (73.2%) | 57 (73.1%) | 0.991 |
| Presence of ≥1 coronary artery CTO, n (%) | 83 (69.7%) | 29 (70.7%) | 54 (69.2%) | 0.865 |
| Number of stents implanted, median (25th and 75th) | 1 (1, 2) | 1 (1, 2.5) | 2 (1, 2) | 0.457 |
| Pre-procedural TIMI flow grade 0, n (%) | 85 (71.4%) | 29 (70.7%) | 56 (71.8%) | 0.903 |
| **Echocardiography** |  |  |  |  |
| EF, median (25th and 75th), % | 0.3 (0.22, 0.41) | 0.26 (0.19, 0.32) | 0.33 (0.23, 0.42) | 0.050 |
| FS, median (25th and 75th), % | 15 (11, 20) | 12.5 (10, 16) | 17 (12, 21) | 0.009 |
| SV, median (25th and 75th), mL | 42 (25.75, 56.25) | 37 (21, 55) | 42 (28, 57) | 0.185 |
| LVEDV, median (25th and 75th), mL | 128 (105, 155) | 125 (88, 157) | 128.5 (108, 153.25) | 0.504 |
| LVESV, median (25th and 75th), mL | 88 (68, 113) | 88 (64, 119) | 86.5 (68.75, 107.5) | 0.935 |
| SI, median (25th and 75th), mL/m² | 24.15 (15.1, 31.55) | 21 (12.83, 31.53) | 24.3 (17.65, 31.63) | 0.274 |
| CO, median (25th and 75th), L/min | 3.2 (2.11, 4.28) | 2.75 (1.78, 4.15) | 3.2 (2.48, 4.33) | 0.114 |
| CI, median (25th and 75th), L/min/m² | 1.8 (1.2, 2.3) | 1.55 (1, 2.33) | 1.8 (1.48, 2.3) | 0.098 |
| Interventricular septal thickness, median (25th and 75th), mm | 0.9 (0.7, 1.1) | 0.9 (0.7, 1.1) | 0.9 (0.7, 1.1) | 0.689 |
| Ventricular aneurysm, n (%) | 12 (10.1%) | 4 (9.8%) | 8 (10.3%) | >0.999 |
| Cardiac perforation, n (%) | 4 (3.4%) | 1 (2.4%) | 3 (3.8%) | >0.999 |
| Chordae tendineae rupture, n (%) | 5 (4.2%) | 0 (0%) | 5 (6.4%) | 0.240 |
| **Laboratory test** |  |  |  |  |
| Biochemical indicators |  |  |  |  |
| ALT, median (25th and 75th), mmol/L | 105 (42, 296) | 124 (38.5, 212) | 98 (42.75, 297.5) | 0.975 |
| AST, median (25th and 75th), mmol/L | 261 (82, 589) | 192 (77.5, 442) | 280.5 (100, 657.75) | 0.314 |
| TBIL, median (25th and 75th), mmol/L | 14.1 (11, 21.3) | 12 (8.4, 16.35) | 16.2 (12.28, 21.73) | 0.001 |
| DBIL, median (25th and 75th), mmol/L | 3.9 (2.4, 6.5) | 3 (1.8, 5.5) | 4.5 (2.6, 8.28) | 0.005 |
| IBIL, median (25th and 75th), mmol/L | 10.2 (7.7, 13.2) | 9 (6.05, 10.25) | 10.8 (8.68, 13.55) | <0.001 |
| Urea, median (25th and 75th), mmol/L | 7.9 (6.21, 12.08) | 7.34 (5.7, 10.77) | 8.55 (6.26, 12.47) | 0.070 |
| Cr, median (25th and 75th), μmol/L | 119.9 (89, 154) | 109 (84.8, 146) | 121 (90.5, 155.53) | 0.210 |
| TP, median (25th and 75th), g/L | 58.4 (50.8, 69.4) | 55.1 (49.3, 68.3) | 61.6 (51.7, 69.45) | 0.215 |
| ALB, median (25th and 75th), g/L | 36 (30.2, 40.3) | 33.3 (28.3, 37.55) | 36.85 (31.08, 40.83) | 0.079 |
| GLB, median (25th and 75th), g/L | 23.4 (19.3, 28.9) | 23.3 (18.85, 28.65) | 24.3 (19.3, 29.8) | 0.750 |
| ALP, median (25th and 75th), U/L | 80.3 (61.1, 98.68) | 81.6 (67.7, 99.65) | 75.9 (59.03, 97.98) | 0.350 |
| GGT, median (25th and 75th), U/L | 42.45 (23.7, 85.43) | 30.9 (20.33, 85.43) | 45 (28.58, 85.85) | 0.128 |
| Glu, median (25th and 75th), mmol/L | 11.06 (7.33, 17.68) | 14.85 (8.67, 21.31) | 9.54 (6.86, 16.72) | 0.016 |
| TC, median (25th and 75th), mmol/L | 3.30 (2.51, 4.11) | 3.36 (2.65, 4.06) | 3.29 (2.43, 4.21) | 0.823 |
| TG, median (25th and 75th), mmol/L | 1.25 (0.85, 1.79) | 1.45 (1.01, 2.35) | 1.24 (0.81, 1.68) | 0.065 |
| HDL, C, median (25th and 75th), mmol/L | 0.81 (0.60, 1.04) | 0.78 (0.54, 0.86) | 0.89 (0.69, 1.08) | 0.013 |
| LDL-C, median (25th and 75th), mmol/L | 2.06 (1.65, 2.85) | 2.19 (1.76, 2.83) | 1.94 (1.62, 2.90) | 0.362 |
| LDH, median (25th and 75th), U/L | 721 (380.31, 1497.5) | 565 (268.5, 1102) | 797.5 (423.01, 1932.75) | 0.070 |
| TyG index, median (25th and 75th) | 1.89 (1.44, 2.53) | 2.29 (1.64, 3.02) | 1.72 (1.37, 2.44) | 0.011 |
| Inflammatory markers |  |  |  |  |
| PCT, median (25th and 75th), ng/mL | 1.2 (0.29, 4.46) | 1.67 (0.42, 4.06) | 0.89 (0.28, 4.84) | 0.476 |
| CRP, median (25th and 75th), mg/L | 12.2 (3.9, 64.4) | 5.0 (1.8, 22.2) | 26.37 (7.1, 84.7) | <0.001 |
| IL-6, median (25th and 75th), pg/mL | 137.5 (73.5, 375.8) | 321 (125.8, 2112.5) | 108.5 (64.0, 194.3) | 0.001 |
| Routine blood test |  |  |  |  |
| WBC, median (25th and 75th), ×10⁹/L | 14.34 (10.6, 18.57) | 15.51 (9.61, 19.12) | 14.28 (11.11, 17.60) | 0.574 |
| RBC, median (25th and 75th), ×10¹²/L | 4.3 (3.83, 4.96) | 4.21 (3.86, 4.79) | 4.37 (3.75, 5.04) | 0.411 |
| HB, median (25th and 75th), g/L | 185 (147, 240) | 190 (153.5, 272.5) | 178.5 (143.5, 238.5) | 0.335 |
| PLT, median (25th and 75th), ×10⁹/L | 136 (114, 154) | 131 (114, 142.5) | 141.5 (113.75, 155.25) | 0.102 |
| NEUT%, median (25th and 75th), % | 86.3 (77.7, 90.2) | 84.2 (72.85, 89.8) | 87.15 (79.15, 90.43) | 0.106 |
| NEUT, median (25th and 75th), ×10⁹/L | 12.08 (8.67, 16.06) | 12.14 (7.82, 17.58) | 11.96 (8.85, 15.51) | 0.889 |
| LYM%, median (25th and 75th), % | 8.3 (6, 17.4) | 10.5 (6.45, 22.8) | 7.55 (5.38, 13.85) | 0.028 |
| LYM, median (25th and 75th), ×10⁹/L | 1.16 (0.79, 2.47) | 1.73 (0.77, 4.01) | 1.11 (0.77, 1.56) | 0.030 |
| MONO%, median (25th and 75th), % | 3.9 (2.6, 5.8) | 2.8 (2.4, 5.6) | 4.45 (3, 5.9) | 0.016 |
| MONO, median (25th and 75th), ×10⁹/L | 0.1 (0, 0.4) | 0.2 (0, 0.85) | 0 (0, 0.1) | <0.001 |
| EO%, median (25th and 75th), % | 0.1 (0, 0.2) | 0.1 (0.1, 0.2) | 0.1 (0, 0.13) | 0.191 |
| EO, median (25th and 75th), ×10⁹/L | 0.57 (0.38, 0.8) | 0.46 (0.32, 0.72) | 0.62 (0.42, 0.84) | 0.047 |
| BA%, median (25th and 75th), % | 0.01 (0, 0.04) | 0.04 (0, 0.14) | 0 (0, 0.02) | <0.001 |
| BA, median (25th and 75th), ×10⁹/L | 0.01 (0, 0.02) | 0.02 (0.01, 0.03) | 0.01 (0, 0.02) | 0.043 |
| L/N, median (25th and 75th) | 0.10 (0.07, 0.22) | 0.13 (0.07, 0.32) | 0.09 (0.06, 0.18) | 0.032 |
| L/M, median (25th and 75th) | 2.1 (1.29, 4.15) | 3.19 (1.85, 8.24) | 1.77 (1.17, 3.75) | 0.002 |
| N/L, median (25th and 75th) | 10.25 (4.51, 14.71) | 7.95 (3.17, 13.96) | 11.62 (5.68, 17) | 0.032 |
| N×PLT/L, median (25th and 75th) | 1849.86 (688.19, 3073.04) | 1247.17 (557.08, 2675.05) | 2020.94 (766.86, 3239.21) | 0.151 |
| PLT/L, median (25th and 75th) | 20.89 (9.32, 34.91) | 15.37 (9.21, 30.17) | 22.84 (9.46, 36.48) | 0.160 |
| CRP/L, median (25th and 75th) | 11.48 (2.77, 58.16) | 6.38 (1.02, 19.36) | 23.98 (3.61, 79.09) | 0.002 |
| CRP/ALB, median (25th and 75th) | 0.38 (0.10, 1.99) | 0.16 (0.07, 0.72) | 0.73 (0.20, 2.43) | 0.003 |
| Arterial blood gas |  |  |  |  |
| PaO2, median (25th and 75th), mmHg | 96.5 (59.2, 207.65) | 118.75 (62.2, 260.25) | 83.5 (59.1, 158.5) | 0.062 |
| PaCO2, median (25th and 75th), mmHg | 32.8 (24.15, 42.13) | 36.65 (28.7, 45.4) | 31.1 (21.55, 39.65) | 0.049 |
| Lac, median (25th and 75th), mmol/L | 10 (7.25, 14.15) | 12.9 (8.1, 15.20) | 9.1 (6.1, 13.3) | 0.016 |
| pH, median (25th and 75th) | 7.23 (7.11, 7.35) | 7.20 (7.00, 7.36) | 7.24 (7.16, 7.35) | 0.175 |
| BE, median (25th and 75th), mmol/L | -13.2 (-19.45, -9.09) | -12.8 (-19.4, -9.15) | -13.4 (-19.8, -9.04) | 0.929 |
| HCO3-, median (25th and 75th), mmol/L | 14.9 (11.35, 18.1) | 14.8 (11.33, 18.45) | 15.1 (11.35, 18) | >0.999 |
| K+, median (25th and 75th), mmol/L | 4.1 (3.6, 4.8) | 3.8 (3.25, 4.68) | 4.15 (3.79, 4.81) | 0.013 |
| Na+, median (25th and 75th), mmol/L | 1.07 (1.0, 1.15) | 1.07 (0.98, 1.15) | 1.07 (1.00, 1.14) | 0.748 |
| Ca2+, median (25th and 75th), mmol/L | 140.4 (137, 144) | 142 (137.55, 146) | 140 (136.68, 142.3) | 0.010 |
| Cl-, median (25th and 75th), mmol/L | 107 (101.5, 109.5) | 106 (101, 109.5) | 107 (102, 109.75) | 0.459 |
| Coagulation profile |  |  |  |  |
| PT, median (25th and 75th), s | 13.9 (12.2, 16.1) | 13.7 (13, 15.25) | 14.05 (12, 16.58) | 0.865 |
| INR, median (25th and 75th) | 1.26 (1.11, 1.47) | 1.23 (1.19, 1.39) | 1.28 (1.10, 1.51) | 0.867 |
| FIB, median (25th and 75th), g/L | 2.72 (2.11, 3.86) | 2.57 (1.9, 3.84) | 2.94 (2.15, 3.89) | 0.160 |
| APTT, median (25th and 75th), s | 34.9 (30.6, 75.5) | 36.5 (30.6, 136.0) | 34.0 (30.6, 68.3) | 0.388 |
| TT, median (25th and 75th), s | 15.6 (13.5, 112.2) | 16.5 (13.6, 150.0) | 15.6 (13.4, 36.8) | 0.309 |
| D-D, median (25th and 75th), mg/L FEU | 3.94 (0.92, 17.51) | 12.98 (0.88, 39.45) | 2.24 (0.92, 6.86) | 0.010 |
| FDP, median (25th and 75th), mg/L | 8.13 (2.15, 23.63) | 14.91 (1.95, 69.14) | 5.94 (2.15, 14.87) | 0.117 |
| **Outcomes** |  |  |  |  |
| ECMO-related complications |  |  |  |  |
| Intracranial haemorrhage, n (%) | 3 (2.5%) | 1 (2.4%) | 2 (2.6%) | >0.999 |
| Gastrointestinal bleeding, n (%) | 13 (10.9%) | 5 (12.2%) | 8 (10.3%) | 0.990 |
| AKI, n (%) | 52 (43.7%) | 16 (39%) | 36 (46.2%) | 0.456 |
| Duration of ECMO support, median (25th and 75th), h | 102.0 (47.0, 152.5) | 58.3 (28.0, 107.3) | 117.5 (72.5, 176.5) | <0.001 |
| Total hospital stay, median (25th and 75th), days | 8 (4, 17) | 4 (1, 12) | 10.5 (4.75, 18.25) | 0.002 |
| ICU stay, median (25th and 75th), days | 7 (4, 14) | 4 (1, 10.5) | 9 (4, 16.25) | 0.001 |
| In-hospital mortality, n (%) | 74 (62.2%) | 32 (78%) | 42 (53.8%) | 0.010 |

BMI, body mass index; PCI, percutaneous coronary intervention; SOFA, SOFA, Sequential Organ Failure Assessment; SAVE, Survival After Veno-arterial ECMO; AMI, acute myocardial infarction; CAG, coronary angiography; LM, left main (coronary artery); LAD, left anterior descending (coronary artery); LCX, left circumflex (coronary artery); RCA, right coronary artery; CTO, chronic total occlusion; TIMI, thrombolysis in myocardial infarction (flow grade); EF, ejection fraction; FS, fractional shortening; SV, stroke volume; LVEDV, left ventricular end-diastolic volume; LVESV, left ventricular end-systolic volume; SI, stroke index; CO, cardiac output; CI, cardiac index; ALT, alanine aminotransferase; AST, aspartate aminotransferase; TBIL, total bilirubin; DBIL, direct bilirubin; IBIL, indirect bilirubin; Urea, blood urea nitrogen; Cr, creatinine; TP, total protein; ALB, albumin; GLB, globulin; ALP, alkaline phosphatase; GGT, gamma-glutamyl transferase; Glu, glucose; TC, total cholesterol; TG, triglyceride; HDL-C, high-density lipoprotein cholesterol; LDL-C, low-density lipoprotein cholesterol; LDH, lactate dehydrogenase; TyG index, triglyceride-glucose index; PCT, procalcitonin; CRP, c-reactive protein; IL-6, interleukin-6; WBC, white blood cell count; RBC, red blood cell count; HB, hemoglobin; PLT, platelet count; NEUT%, neutrophil percentage; NEUT, neutrophil count; LYM%, lymphocyte percentage; LYM, lymphocyte count; MONO%, monocyte percentage; MONO, monocyte count; EO%, eosinophil percentage; EO, eosinophil count; BA%, basophil percentage; BA, basophil count; L/N, lymphocyte-to-neutrophil ratio; L/M, lymphocyte-to-monocyte ratio; N/L, neutrophil-to-lymphocyte ratio; N×PLT/L, neutrophil–platelet–lymphocyte index; PLT/L, platelet-to-lymphocyte ratio; CRP/L, c-reactive protein-to-lymphocyte ratio; CRP/ALB, c-reactive protein-to-albumin ratio; PaO₂, partial pressure of oxygen; PaCO₂, partial pressure of carbon dioxide; Lac, lactate; pH, potential of hydrogen; BE, base excess; HCO₃⁻, bicarbonate; K⁺, potassium; Na⁺, sodium; Ca²⁺, calcium; Cl⁻, chloride; PT, prothrombin time; INR, international normalized ratio; FIB, fibrinogen; APTT, activated partial thromboplastin time; TT, thrombin time; D-D, d-dimer; FDP, fibrin degradation products; ECMO, extracorporeal membrane oxygenation; AKI, acute kidney injury; ICU, intensive care unit.

**Supplementary Table 2.** Lactate levels and dynamic changes.

|  | **Total (n=119)** | **Stage E (n=41)** | **Stage D (n=78)** | ***P*** |
| --- | --- | --- | --- | --- |
| Lac 0 h, median (25th and 75th), mmol/L | 10.00(7.30, 14.4) | 12.90(8.10, 15.20) | 9.15(6.10, 13.40) | 0.016 |
| Lac 6 h, median (25th and 75th), mmol/L | 5.30(2.90, 10.60) | 9.60(4.65, 11.75) | 4.60(2.67, 7.13) | 0.001 |
| Lac 2 4h, median (25th and 75th), mmol/L | 2.45(1.50, 4.48) | 4.40(1.85, 8.65) | 2.20(1.40, 3.40) | 0.003 |
| ΔLac (6 - 0 h), median (25th and 75th), mmol/L | -4.79(-8.47, -1.30) | -4.30(-9.82, -0.30) | -4.95(-8.42, -1.58) | 0.650 |
| ΔLac (24 - 0 h), median (25th and 75th), mmol/L | -7.72(-11.73, -3.20) | -7.57(-14.31, -3.26) | -7.72(-11.60, -3.00) | 0.930 |
| ΔLac (24 - 6 h), median (25th and 75th), mmol/L | -0.20(-3.50, 0.00) | -0.25(-4.48, 0.00) | -0.20(-3.20, 0.00) | 0.674 |

Lac 0 h, lactate level on admission; Lac 6 h, lactate level at 6 h post-PCI; Lac 24 h, lactate level at 24 h post-PCI; ΔLac (6 - 0 h), the difference between lactate levels at 6 h post-PCI and on admission; ΔLac (24 - 0 h), the difference between lactate levels at 24 h post-PCI and on admission; ΔLac (24 - 6 h), the difference between lactate levels at 24 h and 6 h post-PCI.

**Supplementary Table 3.** Adjustment for confounding factors.

|  | **B** | ***P*** | **OR** | **95%CI** |
| --- | --- | --- | --- | --- |
| Age | 0.049 | 0.013 | 1.051 | 1.011-1.092 |
| SCAI Shock Staging (Stage E) | -1.105 | 0.028 | 0.331 | 0.124-0.888 |
| ΔTnI (6 - 0 h) | -0.048 | 0.044 | 0.953 | 0.910-0.999 |
| ΔMyo (24 - 0 h) | 0.002 | 0.014 | 1.002 | 1.000-1.004 |
| Sex | 0.325 | 0.604 | 1.384 | 0.405-4.730 |
| PCI | -0.212 | 0.756 | 0.809 | 0.213-3.069 |

SCAI,Society for Cardiovascular Angiography and Interventions; ΔTnI (6 - 0 h), the difference between the troponin I level at 6 h post-PCI and that on admission; ΔMyo (24 - 0 h), the difference between the myoglobin level at 24 h post-PCI and that on admission; PCI, percutaneous coronary intervention; 95% CI, confidence interval.

**Supplementary Table 4.** Multivariable linear regression results and collinearity diagnostics - regression coefficients.

| **Variable** | **Coefficient (B)** | **Standard Error** | **Standardised Coefficient (Beta)** | **t** | ***P*** | **Tolerance** | **VIF** |
| --- | --- | --- | --- | --- | --- | --- | --- |
| Age (years) | 0.004 | 0.004 | 0.226 | 2.639 | 0.009 | 0.953 | 1.050 |
| SCAI Shock Staging (Stage E) | 0.197 | 0.089 | 0.193 | 2.222 | 0.028 | 0.925 | 1.081 |
| ΔTnI (6 – 0 h) | –0.009 | 0.004 | –0.170 | –2.008 | 0.047 | 0.973 | 1.027 |
| ΔMYO (24 – 0 h) | 0.000 | 0.000 | 0.226 | 2.573 | 0.011 | 0.907 | 1.102 |

SCAI,Society for Cardiovascular Angiography and Interventions; ΔTnI (6 - 0 h), the difference between the troponin I level at 6 h post-PCI and that on admission; ΔMyo (24 - 0 h), the difference between the myoglobin level at 24 h post-PCI and that on admission; VIF, variance inflation factor.

**Supplementary Table 5.** Multivariable linear regression results and collinearity diagnostics - ANOVA for the regression model.

| **Source** | **Sum of Squares** | **df** | **Mean Square** | **F** | ***P*** |
| --- | --- | --- | --- | --- | --- |
| Regression | 5.675 | 4 | 1.419 | 7.251 | <0.001 |
| Residual | 22.308 | 114 | 0.196 | — | — |
| Total | 27.983 | 118 | — | — | — |

df, degrees of freedom.

**Supplementary Table 6.** Multivariable linear regression results and collinearity diagnostics - collinearity diagnostics.

| **Dimension** | **Eigenvalue** | **Condition Index** | **Constant** | **Age** | **SCAI Stage** | **ΔTnI (6 - 0 h)** | **ΔMy (24 - 0 h)** |
| --- | --- | --- | --- | --- | --- | --- | --- |
| 1 | 2.921 | 1.000 | 0.00 | 0.00 | 0.04 | 0.04 | 0.00 |
| 2 | 1.266 | 1.517 | 0.05 | 0.05 | 0.00 | 0.00 | 0.76 |
| 3 | 0.848 | 1.958 | 0.21 | 0.21 | 0.21 | 0.88 | 0.01 |
| 4 | 0.463 | 2.521 | 0.41 | 0.41 | 0.70 | 0.07 | 0.18 |
| 5 | 0.002 | 23.711 | 0.33 | 0.33 | 0.06 | 0.01 | 0.05 |

SCAI,Society for Cardiovascular Angiography and Interventions; ΔTnI (6 - 0 h), the difference between the troponin I level at 6 h post-PCI and that on admission; ΔMyo (24 - 0 h), the difference between the myoglobin level at 24 h post-PCI and that on admission.

**Supplementary Table 7.** Hosmer–lemeshow.

| **Metric** | **Value** |
| --- | --- |
| Chi-square | 4.254 |
| df | 8 |
| *P* | 0.834 |

df, degrees of freedom.

**Supplementary Table 8.** Bootstrap validation (1000 resamples).

| **Variable** | **Bootstrap SE** | **Bootstrap *P* (2-tailed)** | **95% CI** |
| --- | --- | --- | --- |
| Age | 0.025 | 0.018 | 0.013−0.113 |
| SCAI stage (E vs D) | 0.533 | 0.023 | −2.301 to−0.148 |
| ΔTnI (6 – 0 h) | 0.028 | 0.053 | −0.113−0.001 |
| ΔMyo (24 – 0 h) | 0.002 | 0.045 | 0.000−0.006 |

SCAI,Society for Cardiovascular Angiography and Interventions; ΔTnI (6 - 0 h), the difference between the troponin I level at 6 h post-PCI and that on admission; ΔMyo (24 - 0 h), the difference between the myoglobin level at 24 h post-PCI and that on admission; 95% CI, confidence interval.

**Supplementary Table 9.** **Baseline characteristics and clinical profile of patients in SCAI E.**

|  | **Total (n=41)** | **Survivor(n=9)** | **Non-Survivor (n=32)** | ***P*** |
| --- | --- | --- | --- | --- |
| **Demographic** |  |  |  |  |
| Age, median (25th and 75th), years | 68 (54, 72) | 57 (41, 68) | 69 (54, 73) | 0.052 |
| Female, n (%) | 3 (7.3%) | 1 (11.1%) | 30 (93.8%) | 0.535 |
| BMI, median (25th and 75th), kg/m^2^ | 24.17 (22.07, 25.74) | 22.09 (21.22, 25.22) | 24.19 (22.86, 25.97) | 0.312 |
| Smoking, n (%) | 33 (80.5%) | 8 (88.9%) | 25 (78.1%) | 0.807 |
| **Medical history** |  |  |  |  |
| Hypertension, n (%) | 22 (53.7%) | 1 (11.1%) | 21 (65.6%) | 0.012 |
| Hyperlipidemia, n (%) | 2 (4.9%) | 1 (11.1%) | 1 (3.1%) | 0.395 |
| Diabetes mellitus, n (%) | 14 (34.1%) | 3 (33.3%) | 11 (34.4%) | >0.999 |
| Chronic kidney disease, n (%) | 1 (2.4%) | 0 (0.0%) | 1 (3.1%) | >0.999 |
| Coronary artery disease, n (%) | 11 (26.8%) | 2 (22.2%) | 9 (28.1%) | >0.999 |
| Prior PCI, n (%) | 11 (26.8%) | 2 (22.2%) | 9 (28.1%) | >0.999 |
| Atrial fibrillation, n (%) | 2 (4.9%) | 0 (0.0%) | 2 (6.3%) | >0.999 |
| Acute heart failure, n (%) | 0 (0.0%) | 0 (0.0%) | 0 (0.0%) | NA |
| **Admission assessment findings** |  |  |  |  |
| Heart rate, median (25th and 75th), beats/minute | 91 (70, 108) | 85 (60, 95) | 99 (71, 113) | 0.114 |
| Systolic blood pressure, median (25th and 75th), mmHg | 90 (73, 109) | 85 (76, 110) | 91 (72, 110) | >0.999 |
| Diastolic blood pressure, median (25th and 75th), mmHg | 60 (46, 73) | 50 (46, 74) | 61 (46, 73) | 0.924 |
| Mean arterial pressure, median (25th and 75th), mmHg | 69 (59, 86) | 61 (58, 87) | 72 (60, 86) | 0.702 |
| Ventricular tachycardia/fibrillation, n (%) | 22 (53.7%) | 5 (55.6%) | 17 (53.1%) | >0.999 |
| SOFA score, median (25th and 75th) | 11 (8,12) | 9 (6,12) | 11 (8,13) | 0.106 |
| SAVE score, median (25th and 75th) | -10 (-12，-8) | -8 (-11，-5) | -11 (-12，-8) | 0.137 |
| **Treatment after admission** |  |  |  |  |
| Continuous renal replacement therapy, n (%) | 24 (58.5%) | 2 (22.2%) | 22 (68.8%) | 0.034 |
| Invasive mechanical ventilation, n (%) | 38 (92.7%) | 8 (88.9%) | 30 (93.8%) | 0.535 |
| Non-invasive mechanical ventilation, n (%) | 8 (19.5%) | 1 (11.1%) | 7 (21.9%) | 0.807 |
| Intra-aortic balloon pump support, n (%) | 21 (51.2%) | 4 (44.4%) | 17 (53.1%) | 0.934 |
| Temporary pacemaker support, n (%) | 5 (12.2%) | 1 (11.1%) | 4 (12.5%) | >0.999 |
| PCI, n (%) | 37 (90.2%) | 8 (88.9%) | 29 (90.6%) | >0.999 |
| **Onset time of AMI** |  |  |  |  |
| <12 h, n (%) | 26 (63.4%) | 4 (44.4%) | 22 (68.8%) | 0.366 |
| 12–24 h, n (%) | 4 (9.8%) | 1 (11.1%) | 3 (9.4%) |  |
| >24 h, n (%) | 11 (26.8%) | 4 (44.4%) | 7 (21.9%) |  |
| **CAG (stenosis > 50%)** |  |  |  |  |
| **LM**, n (%) | 7 (17.1%) | 1 (11.1%) | 6 (18.8%) | 0.971 |
| LAD, n (%) | 31 (75.6%) | 7 (77.8%) | 24 (75.0%) | >0.999 |
| LCX, n (%) | 30 (73.2%) | 6 (66.7%) | 24 (75.0%) | 0.942 |
| RCA, n (%) | 29 (70.7%) | 4 (44.4%) | 25 (78.1%) | 0.122 |
| **Occluded artery** |  |  |  |  |
| **LM**, n (%) | 2 (4.9%) | 0 (0.0%) | 2 (6.3%) | >0.999 |
| LAD, n (%) | 11 (26.8%) | 1 (11.1%) | 10 (31.3%) | 0.436 |
| LCX, n (%) | 6 (14.6%) | 0 (0.0%) | 6 (18.8%) | 0.383 |
| RCA, n (%) | 16 (39.0%) | 3 (33.3%) | 13 (40.6%) | 0.992 |
| ≥2-vessel coronary artery disease, n (%) | 30 (73.2%) | 5 (55.6%) | 25 (78.1%) | 0.355 |
| Presence of ≥1 coronary artery CTO, n (%) | 29 (70.7%) | 4 (44.4%) | 25 (78.1%) | 0.122 |
| Number of stents implanted, median (25th and 75th) | 1（1,3） | 1 (1,2) | 1 (0,3) | 0.938 |
| Pre-procedural TIMI flow grade 0, n (%) | 29 (70.7%) | 4 (44.4%) | 25 (78.1%) | 0.122 |
| **Echocardiography** |  |  |  |  |
| EF, median (25th and 75th), % | 0.26 (0.19, 0.32) | 0.31 (0.24, 0.51) | 0.25 (0.19, 0.32) | 0.062 |
| FS, median (25th and 75th), % | 12.50 (10.00, 16.00) | 15.00 (10.50, 26.00) | 12.00 (10.00, 16.00) | 0.312 |
| SV, median (25th and 75th), mL | 37.00 (21.00, 55.00) | 46.00 (25.00, 49.50) | 36.50 (18.75, 57.50) | 0.516 |
| LVEDV, median (25th and 75th), mL | 125.00 (88.00, 157.00) | 117.00 (85.50, 147.00) | 132.00 (86.50, 180.75) | 0.403 |
| LVESV, median (25th and 75th), mL | 88.00 (64.00, 119.00) | 79.00 (44.00, 103.50) | 96.50 (66.25, 123.25) | 0.160 |
| SI, median (25th and 75th), mL/m² | 21.00 (12.83, 31.53) | 25.50 (13.80, 29.25) | 20.80 (11.30, 32.45) | 0.788 |
| CO, median (25th and 75th), L/min | 2.75 (1.78, 4.15) | 2.70 (1.95, 4.15) | 2.80 (1.45, 4.20) | 0.730 |
| CI, median (25th and 75th), L/min/m² | 1.55 (1.00, 2.33) | 1.40 (1.05, 2.35) | 1.60 (0.80, 2.30) | 0.730 |
| Interventricular septal thickness, median (25th and 75th), mm | 0.90 (0.70, 1.10) | 1.00 (0.70, 1.10) | 0.90 (0.70, 1.10) | 0.641 |
| Ventricular aneurysm, n (%) | 4 (9.8%) | 1 (11.1%) | 3 (9.4%) | >0.999 |
| Cardiac perforation, n (%) | 1 (2.4%) | 0 (0.0%) | 1 (3.1%) | >0.999 |
| Chordae tendineae rupture, n (%) | 0 (0.0%) | 0 (0.0%) | 0 (0.0%) | NA |
| **Laboratory test** |  |  |  |  |
| Biochemical indicators |  |  |  |  |
| ALT, median (25th and 75th), mmol/L | 124.00 (38.50, 212.00) | 93.00 (49.00, 150.50) | 144.50 (38.25, 379.75) | 0.327 |
| AST, median (25th and 75th), mmol/L | 192.00 (77.50, 442.00) | 126.00 (53.50, 295.50) | 292.00 (78.50, 585.75) | 0.174 |
| TBIL, median (25th and 75th), mmol/L | 12.00 (8.40, 16.35) | 12.00 (7.05, 15.05) | 11.80 (8.35, 17.83) | 0.653 |
| DBIL, median (25th and 75th), mmol/L | 3.00 (1.80, 5.50) | 2.10 (1.15, 4.35) | 3.30 (1.88, 5.58) | 0.145 |
| IBIL, median (25th and 75th), mmol/L | 9.00 (6.05, 10.25) | 9.70 (5.95, 10.80) | 7.90 (6.03, 10.38) | 0.865 |
| Urea, median (25th and 75th), mmol/L | 7.34 (5.70, 10.77) | 7.50 (5.97, 9.20) | 7.19 (5.68, 11.88) | 0.938 |
| Cr, median (25th and 75th), μmol/L | 109.00 (84.80, 146.00) | 105.00 (81.85, 120.25) | 112.00 (83.10, 163.78) | 0.312 |
| TP, median (25th and 75th), g/L | 55.10 (49.30, 68.30) | 51.80 (45.80, 64.70) | 56.00 (50.08, 68.95) | 0.393 |
| ALB, median (25th and 75th), g/L | 33.30 (28.30, 37.55) | 32.40 (27.00, 37.55) | 33.70 (28.53, 37.58) | 0.699 |
| GLB, median (25th and 75th), g/L | 23.30 (18.85, 28.65) | 20.60 (17.75, 23.95) | 24.50 (20.73, 28.90) | 0.106 |
| ALP, median (25th and 75th), U/L | 81.60 (67.70, 99.65) | 62.85 (52.80, 93.00) | 84.60 (70.60, 110.88) | 0.055 |
| GGT, median (25th and 75th), U/L | 30.90 (20.33, 85.43) | 22.65 (18.43, 38.78) | 32.75 (21.30, 94.95) | 0.095 |
| Glu, median (25th and 75th), mmol/L | 14.85 (8.67, 21.31) | 13.36 (7.62, 19.75) | 15.09 (8.67, 22.36) | 0.678 |
| TC, median (25th and 75th), mmol/L | 3.36 (2.65, 4.06) | 3.44 (2.59, 4.68) | 3.36 (2.65, 4.00) | 0.584 |
| TG, median (25th and 75th), mmol/L | 1.45 (1.01, 2.35) | 2.33 (1.21, 3.73) | 1.24 (0.90, 1.79) | 0.036 |
| HDL, C, median (25th and 75th), mmol/L | 0.78 (0.54, 0.86) | 0.79 (0.58, 0.93) | 0.78 (0.54, 0.86) | 0.654 |
| LDL-C, median (25th and 75th), mmol/L | 2.19 (1.76, 2.83) | 2.14 (1.67, 3.04) | 2.23 (1.90, 2.78) | 0.934 |
| LDH, median (25th and 75th), U/L | 565.00 (268.50, 1102.00) | 508.00 (240.25, 810.50) | 648.00 (268.50, 1337.00) | 0.293 |
| TyG index, median (25th and 75th) | 2.29 (1.64, 3.02) | 2.92 (1.64, 3.68) | 2.23 (1.56, 2.80) | 0.278 |
| Inflammatory markers |  |  |  |  |
| PCT, median (25th and 75th), ng/mL | 1.67 (0.42, 4.06) | 0.85 (0.18, 1.78) | 1.79 (1.14, 6.56) | 0.074 |
| CRP, median (25th and 75th), mg/L | 5.00 (1.75, 22.17) | 1.85 (0.29, 32.73) | 5.01 (2.58, 21.58) | 0.480 |
| IL-6, median (25th and 75th), pg/mL | 160.50 (137.50, 794.00) | 137.50 (77.75, 203.75) | 250.50 (137.50, 1722.75) | 0.065 |
| Routine blood test |  |  |  |  |
| WBC, median (25th and 75th), ×10⁹/L | 15.51 (9.61, 19.12) | 17.67 (16.01, 21.11) | 13.55 (8.66, 19.07) | 0.120 |
| RBC, median (25th and 75th), ×10¹²/L | 4.21 (3.86, 4.79) | 4.24 (3.95, 4.42) | 4.15 (3.83, 4.89) | 0.793 |
| HB, median (25th and 75th), g/L | 131.00 (114.00, 142.50) | 133.00 (118.00, 139.00) | 130.50 (107.75, 151.50) | 0.722 |
| PLT, median (25th and 75th), ×10⁹/L | 190.00 (153.50, 272.50) | 222.00 (179.50, 319.50) | 187.00 (138.00, 231.75) | 0.106 |
| NEUT%, median (25th and 75th), % | 84.20 (72.85, 89.80) | 78.90 (64.90, 88.60) | 84.85 (75.73, 90.08) | 0.525 |
| NEUT, median (25th and 75th), ×10⁹/L | 12.14 (7.82, 17.58) | 15.83 (9.65, 18.14) | 11.55 (6.89, 17.34) | 0.242 |
| LYM%, median (25th and 75th), % | 10.50 (6.45, 22.80) | 17.50 (4.90, 28.55) | 10.45 (6.60, 21.85) | 0.889 |
| LYM, median (25th and 75th), ×10⁹/L | 1.73 (0.77, 4.01) | 2.85 (0.89, 6.05) | 1.55 (0.75, 3.66) | 0.376 |
| MONO%, median (25th and 75th), % | 2.80 (2.40, 5.60) | 4.70 (2.60, 6.75) | 2.70 (2.33, 4.88) | 0.120 |
| MONO, median (25th and 75th), ×10⁹/L | 0.46 (0.32, 0.72) | 0.75 (0.61, 1.16) | 0.44 (0.25, 0.58) | 0.003 |
| EO%, median (25th and 75th), % | 0.20 (0.00, 0.85) | 0.30 (0.00, 0.90) | 0.20 (0.03, 0.88) | 0.963 |
| EO, median (25th and 75th), ×10⁹/L | 0.04 (0.00, 0.14) | 0.06 (0.00, 0.14) | 0.03 (0.00, 0.14) | 0.793 |
| BA%, median (25th and 75th), % | 0.10 (0.10, 0.20) | 0.10 (0.05, 0.20) | 0.10 (0.10, 0.20) | 0.841 |
| BA, median (25th and 75th), ×10⁹/L | 0.02 (0.01, 0.03) | 0.02 (0.01, 0.04) | 0.02 (0.01, 0.03) | 0.312 |
| L/N, median (25th and 75th) | 0.13 (0.07, 0.32) | 0.22 (0.06, 0.44) | 0.12 (0.07, 0.29) | 0.816 |
| L/M, median (25th and 75th) | 3.19 (1.85, 8.24) | 4.15 (1.21, 7.03) | 3.08 (1.93, 10.30) | 0.566 |
| N/L, median (25th and 75th) | 7.95 (3.17, 13.96) | 4.51 (2.28, 19.12) | 8.02 (3.46, 13.66) | 0.816 |
| N×PLT/L, median (25th and 75th) | 1247.17 (557.08, 2675.05) | 947.34 (533.41, 4933.94) | 1427.70 (536.56, 2612.53) | 0.988 |
| PLT/L, median (25th and 75th) | 15.37 (9.21, 30.17) | 12.00 (7.93, 55.32) | 16.55 (9.16, 29.11) | 0.841 |
| CRP/L, median (25th and 75th) | 6.38 (1.02, 19.36) | 7.12 (0.17, 26.39) | 6.38 (1.11, 16.76) | 0.912 |
| CRP/ALB, median (25th and 75th) | 0.16 (0.07, 0.72) | 0.33 (0.04, 0.90) | 0.16 (0.08, 0.67) | 0.843 |
| Arterial blood gas |  |  |  |  |
| PaO2, median (25th and 75th), mmHg | 118.75 (62.20, 260.25) | 113.00 (73.80, 336.15) | 120.00 (57.70, 262.00) | 0.110 |
| PaCO2, median (25th and 75th), mmHg | 36.65 (28.70, 45.40) | 32.20 (29.30, 35.80) | 38.60 (27.25, 50.23) | 0.387 |
| Lac, median (25th and 75th), mmol/L | 12.90 (8.10, 15.20) | 8.00 (4.15, 17.45) | 13.13 (10.00, 15.26) | 0.018 |
| pH, median (25th and 75th) | 7.20 (7.00, 7.36) | 7.26 (7.16, 7.40) | 7.14 (6.98, 7.33) | 0.018 |
| BE, median (25th and 75th), mmol/L | -12.80 (-19.40, -9.15) | -11.10 (-13.05, -8.15) | -14.20 (-19.48, -8.95) | 0.022 |
| HCO3-, median (25th and 75th), mmol/L | 14.80 (11.33, 18.45) | 16.80 (15.35, 19.55) | 14.50 (10.70, 17.70) | 0.139 |
| K+, median (25th and 75th), mmol/L | 3.80 (3.25, 4.68) | 3.60 (3.25, 5.10) | 3.83 (3.25, 4.56) | 0.159 |
| Na+, median (25th and 75th), mmol/L | 142.00 (137.55, 146.00) | 142.00 (138.00, 145.60) | 142.90 (137.38, 146.60) | 0.680 |
| Ca2+, median (25th and 75th), mmol/L | 1.07 (0.98, 1.15) | 1.00 (0.95, 1.22) | 1.07 (1.00, 1.15) | 0.519 |
| Cl-, median (25th and 75th), mmol/L | 106.00 (101.00, 109.50) | 108.00 (101.00, 111.00) | 105.50 (101.00, 109.75) | 0.840 |
| Coagulation profile |  |  |  |  |
| PT, median (25th and 75th), s | 13.70 (13.00, 15.25) | 13.30 (12.20, 14.35) | 14.15 (13.20, 15.80) | 0.137 |
| INR, median (25th and 75th) | 1.23 (1.19, 1.39) | 1.21 (1.11, 1.30) | 1.29 (1.19, 1.44) | 0.113 |
| FIB, median (25th and 75th), g/L | 2.57 (1.90, 3.84) | 2.39 (2.01, 4.31) | 2.60 (1.78, 3.66) | 0.889 |
| APTT, median (25th and 75th), s | 36.50 (30.60, 136.00) | 54.10 (33.60, 175.05) | 34.55 (29.15, 118.80) | 0.255 |
| TT, median (25th and 75th), s | 16.50 (13.55, 150.00) | 83.70 (20.80, 175.00) | 15.55 (13.28, 150.00) | 0.184 |
| D-D, median (25th and 75th), mg/L FEU | 12.98 (0.88, 39.45) | 10.36 (0.73, 36.54) | 12.98 (0.88, 39.45) | 0.505 |
| FDP, median (25th and 75th), mg/L | 14.91 (1.95, 69.14) | 21.59 (2.10, 115.18) | 14.91 (1.95, 69.14) | 0.983 |
| **Outcomes** |  |  |  |  |
| ECMO-related complications |  |  |  |  |
| Intracranial haemorrhage, n (%) | 1 (2.4%) | 0 (0.0%) | 1 (3.1%) | >0.999 |
| Gastrointestinal bleeding, n (%) | 5 (12.2%) | 0 (0.0%) | 5 (15.6%) | 0.491 |
| AKI, n (%) | 16 (39.0%) | 0 (0.0%) | 16 (50.0%) | 0.020 |
| Duration of ECMO support, median (25th and 75th), h | 58.3 (28.0, 107.3) | 87.0 (58.0, 123.5) | 37.2 (20.0, 102.8) | 0.092 |
| Total hospital stay, median (25th and 75th), days | 4 (1, 12) | 16.00 (12, 23) | 4 (1, 6) | <0.001 |
| ICU stay, median (25th and 75th), days | 4 (1, 11) | 13 (11, 17) | 3 (1, 6) | <0.001 |

BMI, body mass index; PCI, percutaneous coronary intervention; SOFA, SOFA, Sequential Organ Failure Assessment; SAVE, Survival After Veno-arterial ECMO; AMI, acute myocardial infarction; CAG, coronary angiography; LM, left main (coronary artery); LAD, left anterior descending (coronary artery); LCX, left circumflex (coronary artery); RCA, right coronary artery; CTO, chronic total occlusion; TIMI, thrombolysis in myocardial infarction (flow grade); EF, ejection fraction; FS, fractional shortening; SV, stroke volume; LVEDV, left ventricular end-diastolic volume; LVESV, left ventricular end-systolic volume; SI, stroke index; CO, cardiac output; CI, cardiac index; ALT, alanine aminotransferase; AST, aspartate aminotransferase; TBIL, total bilirubin; DBIL, direct bilirubin; IBIL, indirect bilirubin; Urea, blood urea nitrogen; Cr, creatinine; TP, total protein; ALB, albumin; GLB, globulin; ALP, alkaline phosphatase; GGT, gamma-glutamyl transferase; Glu, glucose; TC, total cholesterol; TG, triglyceride; HDL-C, high-density lipoprotein cholesterol; LDL-C, low-density lipoprotein cholesterol; LDH, lactate dehydrogenase; TyG index, triglyceride-glucose index; PCT, procalcitonin; CRP, c-reactive protein; IL-6, interleukin-6; WBC, white blood cell count; RBC, red blood cell count; HB, hemoglobin; PLT, platelet count; NEUT%, neutrophil percentage; NEUT, neutrophil count; LYM%, lymphocyte percentage; LYM, lymphocyte count; MONO%, monocyte percentage; MONO, monocyte count; EO%, eosinophil percentage; EO, eosinophil count; BA%, basophil percentage; BA, basophil count; L/N, lymphocyte-to-neutrophil ratio; L/M, lymphocyte-to-monocyte ratio; N/L, neutrophil-to-lymphocyte ratio; N×PLT/L, neutrophil–platelet–lymphocyte index; PLT/L, platelet-to-lymphocyte ratio; CRP/L, c-reactive protein-to-lymphocyte ratio; CRP/ALB, c-reactive protein-to-albumin ratio; PaO₂, partial pressure of oxygen; PaCO₂, partial pressure of carbon dioxide; Lac, lactate; pH, potential of hydrogen; BE, base excess; HCO₃⁻, bicarbonate; K⁺, potassium; Na⁺, sodium; Ca²⁺, calcium; Cl⁻, chloride; PT, prothrombin time; INR, international normalized ratio; FIB, fibrinogen; APTT, activated partial thromboplastin time; TT, thrombin time; D-D, d-dimer; FDP, fibrin degradation products; ECMO, extracorporeal membrane oxygenation; AKI, acute kidney injury; ICU, intensive care unit.

**Supplementary Table 10.** Cardiac biomarkers and dynamic changes in SCAI E.

|  | **Total (n=41)** | **Survivor(n=9)** | **Non-Survivor (n=32)** | ***P*** |
| --- | --- | --- | --- | --- |
| **Hospital admission** |  |  |  |  |
| TnI, median (25th and 75th), ng/mL | 1.45 (0.49, 7.33) | 0.95 (0.26, 2.25) | 1.80 (0.51, 7.80) | 0.152 |
| CK-MB, median (25th and 75th), ng/mL | 53.50 (17.25, 246.75) | 21.00 (9.45, 75.00) | 75.00 (24.00, 337.00) | 0.107 |
| Myo, median (25th and 75th), ng/mL | 900.00 (771.50, 900.00) | 900.00 (519.50, 900.00) | 900.00 (812.50, 900.00) | 0.736 |
| NT-proBNP, median (25th and 75th), pg/mL | 2145.00 (397.50, 5597.50) | 363.00 (143.50, 2470.00) | 2630.00 (443.50, 6135.00) | 0.049 |
| D-dimer, median (25th and 75th), ng/mL | 10515.00 (1064.50, 35050.00) | 3060.00 (579.00, 18250.00) | 14900.00 (1180.00, 41800.00) | 0.234 |
| **6 h postoperatively** |  |  |  |  |
| TnI, median (25th and 75th), ng/mL | 22.00 (3.30, 25.00) | 16.00 (1.20, 25.00) | 22.00 (4.65, 25.00) | 0.420 |
| CK-MB, median (25th and 75th), ng/mL | 500.00 (96.00, 500.00) | 264.00 (29.00, 500.00) | 500.00 (124.50, 500.00) | 0.532 |
| Myo, median (25th and 75th), ng/mL | 900.00 (900.00, 900.00) | 900.00 (404.00, 900.00) | 900.00 (900.00, 900.00) | 0.161 |
| **24 h postoperatively** |  |  |  |  |
| TnI, median (25th and 75th), ng/mL | 11.00 (3.05, 25.00) | 4.00 (0.95, 11.00) | 13.50 (5.88, 25.00) | 0.062 |
| CK-MB, median (25th and 75th), ng/mL | 258.00 (78.00, 500.00) | 123.00 (33.00, 331.00) | 293.50 (115.00, 500.00) | 0.165 |
| Myo, median (25th and 75th), ng/mL | 900.00 (900.00, 900.00) | 900.00 (760.00, 900.00) | 900.00 (900.00, 900.00) | 0.469 |
| NT-proBNP, median (25th and 75th), pg/mL | 2890.00 (1010.00, 5460.00) | 1380.00 (276.50, 2167.50) | 3460.00 (2220.00, 5980.00) | 0.014 |
| D-dimer,, median (25th and 75th), ng/mL | 4145.00 (1244.25, 17000.00) | 6838.50 (777.00, 0.00) | 4145.00 (1475.00, 24800.00) | 0.889 |
| ΔTnI (6 - 0 h), median (25th and 75th), mmol/L | 7.90 (1.19, 22.70) | 15.05 (1.19, 23.70) | 7.15 (0.76, 21.11) | 0.627 |
| ΔCK-MB (6 h - 0 h), median (25th and 75th), mmol/L | 119.00 (8.00, 414.00) | 249.00 (10.30, 427.00) | 112.00 (1.88, 285.50) | 0.317 |
| ΔMyo (6 - 0 h), median (25th and 75th), mmol/L | 0.00 (0.00, 40.00) | 0.00 (0.00, 40.00) | 0.00 (0.00, 63.75) | 0.595 |
| ΔTnI (24 - 6 h), median (25th and 75th), mmol/L | 0.00 (-4.00, 0.10) | -0.43 (-8.75, 0.03) | 0.00 (-3.50, 0.67) | 0.216 |
| ΔCK-MB (24 - 6 h), median (25th and 75th), mmol/L | -1.40 (-81.00, 0.00) | -4.00 (-180.75, 12.00) | -1.40 (-71.00, 0.00) | 0.932 |
| ΔMyo (24 - 6 h), median (25th and 75th), mmol/L | 0.00 (0.00, 0.00) | 0.00 (-174.50, 476.25) | 0.00 (0.00, 0.00) | 0.977 |
| ΔTnI (24 - 0 h), median (25th and 75th), mmol/L | 5.10 (1.03, 18.90) | 1.33 (0.26, 10.05) | 10.30 (1.66, 21.10) | 0.189 |
| ΔCK-MB (24 - 0 h), median (25th and 75th), mmol/L | 101.50 (-1.60, 379.25) | 33.00 (0.00, 254.00) | 105.00 (-13.35, 419.50) | 0.678 |
| ΔMyo (24 - 0 h), median (25th and 75th), mmol/L | 0.00 (0.00, 93.25) | 0.00 (-140.00, 0.00) | 0.00 (0.00, 162.00) | 0.249 |
| ΔNT-proBNP (24 - 0 h), median (25th and 75th), mmol/L | 707.00 (-1550.50, 2002.50) | 590.00 (-1411.50, 1426.25) | 707.00 (-4210.00, 3240.75) | 0.820 |
| ΔD-dimer, (24 - 0 h), median (25th and 75th), mmol/L | -79.00 (-35845.00, 2261.00) | 5005.50 (-2283.00, 0.00) | -79.00 (-39900.00, 510.00) | 0.500 |

TnI, troponin I; CK-MB, creatine kinase–MB isoenzyme; Myo, myoglobin; NT-proBNP, n-terminal pro–b-type natriuretic peptide; Lac, lactate; ΔTnI (6 - 0 h), the difference between the troponin I level at 6 h post-pci and that on admission (0 h); ΔCK-MB (6 - 0 h), the difference between the creatine kinase–mb isoenzyme level at 6 h post-pci and that on admission; ΔMyo (6 - 0 h), the difference between the myoglobin level at 6 h post-pci and that on admission; ΔTnI (24 - 6 h), the difference between the troponin I level at 24 h and 6 h post-pci; ΔCK-MB (24 - 6 h), the difference between the creatine kinase–mb isoenzyme level at 24 h and 6 h post-pci; ΔMyo (24 - 6 h), the difference between the myoglobin level at 24 h and 6 h post-pci; ΔTnI (24 - 0 h), the difference between the troponin I level at 24 h post-pci and that on admission; ΔCK-MB (24 - 0 h), the difference between the creatine kinase–mb isoenzyme level at 24 h post-pci and that on admission; ΔMyo (24 - 0 h), the difference between the myoglobin level at 24 h post-pci and that on admission; ΔNT-proBNP (24 - 0 h), the difference between the n-terminal pro–b-type natriuretic peptide level at 24 h post-pci and that on admission; ΔD-Dimer (24 - 0 h), the difference between the d-dimer level at 24 h post-pci and that on admission.

**Supplementary Table 11.** Lactate levels and dynamic changes in SCAI E.

|  | **Total (n=41)** | **Survivor(n=9)** | **Non-Survivor (n=32)** | ***P*** |
| --- | --- | --- | --- | --- |
| Lac 0 h, median (25th and 75th), mmol/L | 12.90 (8.10, 15.20) | 8.00 (4.15, 17.45) | 13.13 (10.00, 15.26) | 0.218 |
| Lac 6 h, median (25th and 75th), mmol/L | 9.60 (4.65, 11.75) | 3.80 (2.70, 7.60) | 10.35 (6.10, 12.85) | 0.048 |
| Lac 2 4h, median (25th and 75th), mmol/L | 4.40 (1.85, 8.65) | 1.70 (1.30, 4.40) | 6.45 (3.20, 11.90) | 0.017 |
| ΔLac (6 - 0 h), median (25th and 75th), mmol/L | -4.30 (-9.82, -0.30) | -2.40 (-13.52, -0.35) | -4.75 (-10.14, -0.03) | 0.987 |
| ΔLac (24 - 0 h), median (25th and 75th), mmol/L | -7.57 (-14.31, -3.26) | -6.30 (-14.82, -1.55) | -7.84 (-14.48, -3.42) | 0.693 |
| ΔLac (24 - 6 h), median (25th and 75th), mmol/L | -0.25 (-4.48, 0.00) | -1.40 (-5.70, 0.00) | -0.20 (-4.10, 0.00) | 0.517 |

Lac 0 h, lactate level on admission; Lac 6 h, lactate level at 6 h post-PCI; Lac 24 h, lactate level at 24 h post-PCI; ΔLac (6 - 0 h), the difference between lactate levels at 6 h post-PCI and on admission; ΔLac (24 - 0 h), the difference between lactate levels at 24 h post-PCI and on admission; ΔLac (24 - 6 h), the difference between lactate levels at 24 h and 6 h post-PCI.

**Supplementary Table 12.** **Baseline characteristics and clinical profile of patients in SCAI D.**

|  | **Total (n=78)** | **Survivor（n=36）** | **Non-Survivor(n=42)** | ***P*** |
| --- | --- | --- | --- | --- |
| **Demographic** |  |  |  |  |
| Age, median (25th and 75th), years | 61 (54, 69) | 59 (50, 67) | 64 (56, 70) | 0.030 |
| Female, n (%) | 15 (19.2%) | 5 (13.9%) | 10 (23.8%) | 0.268 |
| BMI, median (25th and 75th), kg/m^2^ | 24 (22, 26) | 23 (21, 26) | 24 (22, 26) | 0.696 |
| Smoking, n (%) | 57 (73.1%) | 29 (80.6%) | 28 (66.7%) | 0.168 |
| **Medical history** |  |  |  |  |
| Hypertension, n (%) | 38 (48.7%) | 21 (58.3%) | 17 (40.5%) | 0.116 |
| Hyperlipidemia, n (%) | 2 (2.6%) | 0 (0.0%) | 2 (4.8%) | 0.543 |
| Diabetes mellitus, n (%) | 25 (32.1%) | 11 (30.6%) | 14 (33.3%) | 0.798 |
| Chronic kidney disease, n (%) | 0 (0.0%) | 0 (0.0%) | 0 (0.0%) | NA |
| Coronary artery disease, n (%) | 22 (28.2%) | 9 (25.0%) | 13 (31.0%) | 0.560 |
| Prior PCI, n (%) | 18 (23.1%) | 8 (22.2%) | 10 (23.8%) | 0.868 |
| Atrial fibrillation, n (%) | 0 (0.0%) | 0 (0.0%) | 0 (0.0%) | NA |
| Acute heart failure, n (%) | 2 (2.6%) | 0 (0.0%) | 2 (4.8%) | 0.497 |
| **Admission assessment findings** |  |  |  |  |
| Heart rate, median (25th and 75th), beats/minute | 93 (73, 108) | 93 (81, 101) | 92 (69, 111) | 0.771 |
| Systolic blood pressure, median (25th and 75th), mmHg | 105 (86, 121) | 105 (91, 120) | 104 (84, 125) | 0.693 |
| Diastolic blood pressure, median (25th and 75th), mmHg | 67 (54, 80) | 67 (56, 79) | 65 (53, 82) | 0.700 |
| Mean arterial pressure, median (25th and 75th), mmHg | 79 (69, 93) | 81 (71, 93) | 77 (67, 100) | 0.647 |
| Ventricular tachycardia/fibrillation, n (%) | 27 (34.6%) | 12 (33.3%) | 15 (35.7%) | 0.826 |
| SOFA score, median (25th and 75th) | 9 (8, 11) | 8 (6, 11) | 10 (9, 12) | 0.035 |
| SAVE score, median (25th and 75th) | -2 (-7, 1) | -1 (-3, 2) | -5 (-8, -1) | 0.001 |
| **Treatment after admission** |  |  |  |  |
| Continuous renal replacement therapy, n (%) | 37 (47.4%) | 9 (25.0%) | 28 (66.7%) | <0.001 |
| Invasive mechanical ventilation, n (%) | 46 (59.0%) | 15 (41.7%) | 31 (73.8%) | 0.004 |
| Non-invasive mechanical ventilation, n (%) | 20 (25.6%) | 6 (16.7%) | 14 (33.3%) | 0.093 |
| Intra-aortic balloon pump support, n (%) | 44 (56.4%) | 20 (55.6%) | 24 (57.1%) | 0.888 |
| Temporary pacemaker support, n (%) | 14 (17.9%) | 7 (19.4%) | 7 (16.7%) | 0.750 |
| PCI, n (%) | 70 (89.7%) | 32 (88.9%) | 38 (90.5%) | 0.818 |
| **Onset time of AMI** |  |  |  |  |
| <12 h, n (%) | 29 (37.2%) | 16 (44.4%) | 13 (31.0%) | 0.400 |
| 12–24 h, n (%) | 17 (21.8%) | 6 (16.7%) | 11 (26.2%) |  |
| >24 h, n (%) | 32 (41.0%) | 14 (38.9%) | 18 (42.9%) |  |
| **CAG (stenosis > 50%)** |  |  |  |  |
| **LM**, n (%) | 12 (15.4%) | 7 (19.4%) | 5 (11.9%) | 0.358 |
| LAD, n (%) | 55 (70.5%) | 27 (75.0%) | 28 (66.7%) | 0.421 |
| LCX, n (%) | 43 (55.1%) | 19 (52.8%) | 24 (57.1%) | 0.699 |
| RCA, n (%) | 48 (61.5%) | 26 (72.2%) | 22 (52.4%) | 0.073 |
| **Occluded artery** |  |  |  |  |
| **LM**, n (%) | 6 (7.7%) | 4 (11.1%) | 2 (4.8%) | 0.533 |
| LAD, n (%) | 28 (35.9%) | 16 (44.4%) | 12 (28.6%) | 0.145 |
| LCX, n (%) | 13 (16.7%) | 5 (13.9%) | 8 (19.0%) | 0.542 |
| RCA, n (%) | 24 (30.8%) | 14 (38.9%) | 10 (23.8%) | 0.150 |
| ≥2-vessel coronary artery disease, n (%) | 57 (73.1%) | 26 (72.2%) | 31 (73.8%) | 0.875 |
| Presence of ≥1 coronary artery CTO, n (%) | 54 (69.2%) | 28 (77.8%) | 26 (61.9%) | 0.130 |
| Number of stents implanted, median (25th and 75th) | 2 (1, 2) | 2 (1, 2) | 1 (0, 3) | 0.610 |
| Pre-procedural TIMI flow grade 0, n (%) | 56 (71.8%) | 29 (80.6%) | 27 (64.3%) | 0.111 |
| **Echocardiography** |  |  |  |  |
| EF, median (25th and 75th), % | 0.33 (0.23, 0.42) | 0.33 (0.26, 0.43) | 0.33 (0.22, 0.42) | 0.603 |
| FS, median (25th and 75th), % | 17.00 (12.00, 21.00) | 17.00 (13.00, 21.00) | 17.00 (11.00, 21.00) | 0.683 |
| SV, median (25th and 75th), mL | 42.00 (28.00, 57.00) | 45.00 (35.75, 54.00) | 39.00 (24.00, 64.00) | 0.497 |
| LVEDV, median (25th and 75th), mL | 128.50 (108.00, 153.25) | 129.50 (114.00, 153.50) | 121.00 (102.50, 155.00) | 0.52 |
| LVESV, median (25th and 75th), mL | 86.50 (68.75, 107.50) | 85.00 (69.25, 113.75) | 87.50 (68.75, 107.50) | 0.88 |
| SI, median (25th and 75th), mL/m² | 24.30 (17.65, 31.63) | 24.40 (20.73, 29.93) | 22.95 (14.50, 34.33) | 0.516 |
| CO, median (25th and 75th), L/min | 3.20 (2.48, 4.33) | 3.21 (2.90, 3.80) | 3.20 (2.13, 5.00) | 0.987 |
| CI, median (25th and 75th), L/min/m² | 1.80 (1.48, 2.30) | 1.85 (1.70, 2.11) | 1.80 (1.18, 2.70) | 0.931 |
| Interventricular septal thickness, median (25th and 75th), mm | 0.90 (0.70, 1.10) | 0.90 (0.70, 1.18) | 0.90 (0.70, 1.10) | 0.514 |
| Ventricular aneurysm, n (%) | 8 (10.3%) | 2 (5.6%) | 6 (14.3%) | 0.372 |
| Cardiac perforation, n (%) | 3 (3.8%) | 0 (0.0%) | 3 (7.1%) | 0.296 |
| Chordae tendineae rupture, n (%) | 5 (6.4%) | 0 (0.0%) | 5 (11.9%) | 0.094 |
| **Laboratory test** |  |  |  |  |
| Biochemical indicators |  |  |  |  |
| ALT, median (25th and 75th), mmol/L | 98.00 (42.75, 297.50) | 106.00 (42.75, 229.00) | 97.00 (42.75, 338.00) | 0.787 |
| AST, median (25th and 75th), mmol/L | 280.50 (100.00, 657.75) | 329.00 (120.50, 723.00) | 193.00 (79.50, 643.50) | 0.578 |
| TBIL, median (25th and 75th), mmol/L | 16.20 (12.28, 21.73) | 14.25 (11.68, 20.95) | 18.25 (12.45, 21.95) | 0.245 |
| DBIL, median (25th and 75th), mmol/L | 4.50 (2.60, 8.28) | 2.90 (2.43, 7.05) | 4.90 (2.98, 8.50) | 0.093 |
| IBIL, median (25th and 75th), mmol/L | 10.80 (8.68, 13.55) | 10.70 (8.23, 13.48) | 11.20 (9.08, 15.20) | 0.685 |
| Urea, median (25th and 75th), mmol/L | 8.55 (6.26, 12.47) | 9.18 (6.39, 11.91) | 8.42 (6.23, 12.71) | 0.7 |
| Cr, median (25th and 75th), μmol/L | 121.00 (90.50, 155.53) | 120.70 (91.58, 154.75) | 123.65 (88.50, 193.35) | 0.833 |
| TP, median (25th and 75th), g/L | 61.60 (51.70, 69.45) | 60.85 (53.35, 66.20) | 64.15 (47.88, 71.23) | 0.822 |
| ALB, median (25th and 75th), g/L | 36.85 (31.08, 40.83) | 37.25 (32.03, 42.88) | 36.10 (28.80, 39.93) | 0.107 |
| GLB, median (25th and 75th), g/L | 24.30 (19.30, 29.80) | 22.30 (19.58, 27.08) | 26.60 (18.73, 32.33) | 0.208 |
| ALP, median (25th and 75th), U/L | 75.90 (59.03, 97.98) | 72.10 (59.00, 96.00) | 82.60 (59.15, 100.60) | 0.498 |
| GGT, median (25th and 75th), U/L | 45.00 (28.58, 85.85) | 44.30 (28.90, 84.50) | 46.70 (28.15, 90.80) | 0.946 |
| Glu, median (25th and 75th), mmol/L | 9.54 (6.86, 16.72) | 8.97 (6.74, 17.13) | 10.91 (6.88, 16.50) | 0.532 |
| TC, median (25th and 75th), mmol/L | 3.29 (2.43, 4.21) | 3.43 (2.64, 4.53) | 3.20 (2.28, 3.96) | 0.274 |
| TG, median (25th and 75th), mmol/L | 1.24 (0.81, 1.68) | 1.37 (0.82, 1.79) | 1.12 (0.78, 1.43) | 0.223 |
| HDL, C, median (25th and 75th), mmol/L | 0.89 (0.69, 1.08) | 0.89 (0.75, 1.08) | 0.89 (0.57, 1.10) | 0.505 |
| LDL-C, median (25th and 75th), mmol/L | 1.94 (1.62, 2.90) | 2.05 (1.66, 3.21) | 1.93 (1.46, 2.55) | 0.256 |
| LDH, median (25th and 75th), U/L | 797.50 (423.01, 1932.75) | 848.00 (412.68, 1857.00) | 756.00 (428.50, 1982.00) | 0.921 |
| TyG index, median (25th and 75th) | 1.72 (1.37, 2.44) | 1.89 (1.41, 2.45) | 1.69 (1.31, 2.47) | 0.681 |
| Inflammatory markers |  |  |  |  |
| PCT, median (25th and 75th), ng/mL | 0.89 (0.28, 4.84) | 0.83 (0.25, 4.71) | 0.95 (0.36, 6.00) | 0.575 |
| CRP, median (25th and 75th), mg/L | 26.37 (7.10, 84.68) | 12.89 (3.54, 67.19) | 32.83 (10.18, 97.45) | 0.181 |
| IL-6, median (25th and 75th), pg/mL | 137.50 (77.50, 142.50) | 125.50 (49.18, 137.50) | 137.50 (99.85, 194.25) | 0.007 |
| Routine blood test |  |  |  |  |
| WBC, median (25th and 75th), ×10⁹/L | 14.28 (11.11, 17.60) | 13.81 (10.95, 17.10) | 14.34 (11.01, 18.30) | 0.524 |
| RBC, median (25th and 75th), ×10¹²/L | 4.37 (3.75, 5.04) | 4.47 (3.86, 5.18) | 4.31 (3.62, 5.04) | 0.458 |
| HB, median (25th and 75th), g/L | 141.50 (113.75, 155.25) | 143.00 (127.75, 157.75) | 137.50 (112.50, 155.25) | 0.634 |
| PLT, median (25th and 75th), ×10⁹/L | 178.50 (143.50, 238.50) | 197.00 (153.00, 239.50) | 172.00 (117.75, 236.75) | 0.257 |
| NEUT%, median (25th and 75th), % | 87.15 (79.15, 90.43) | 88.00 (82.45, 90.40) | 86.30 (78.58, 90.55) | 0.489 |
| NEUT, median (25th and 75th), ×10⁹/L | 11.96 (8.85, 15.51) | 11.66 (9.65, 15.38) | 12.10 (8.48, 16.08) | 0.752 |
| LYM%, median (25th and 75th), % | 7.55 (5.38, 13.85) | 7.15 (5.83, 11.23) | 8.65 (5.28, 14.63) | 0.571 |
| LYM, median (25th and 75th), ×10⁹/L | 1.11 (0.77, 1.56) | 1.07 (0.72, 1.38) | 1.19 (0.92, 1.58) | 0.378 |
| MONO%, median (25th and 75th), % | 4.45 (3.00, 5.90) | 4.15 (3.00, 5.70) | 4.55 (3.13, 6.10) | 0.578 |
| MONO, median (25th and 75th), ×10⁹/L | 0.62 (0.42, 0.84) | 0.55 (0.36, 0.78) | 0.68 (0.50, 0.88) | 0.219 |
| EO%, median (25th and 75th), % | 0.00 (0.00, 0.10) | 0.00 (0.00, 0.10) | 0.00 (0.00, 0.20) | 0.83 |
| EO, median (25th and 75th), ×10⁹/L | 0.00 (0.00, 0.02) | 0.00 (0.00, 0.02) | 0.00 (0.00, 0.02) | 0.938 |
| BA%, median (25th and 75th), % | 0.10 (0.00, 0.13) | 0.10 (0.00, 0.10) | 0.10 (0.00, 0.20) | 0.903 |
| BA, median (25th and 75th), ×10⁹/L | 0.01 (0.00, 0.02) | 0.01 (0.00, 0.02) | 0.01 (0.00, 0.02) | 0.867 |
| L/N, median (25th and 75th) | 0.09 (0.06, 0.18) | 0.08 (0.07, 0.14) | 0.10 (0.06, 0.19) | 0.528 |
| L/M, median (25th and 75th) | 1.77 (1.17, 3.75) | 1.77 (1.12, 3.62) | 1.80 (1.17, 3.90) | 0.896 |
| N/L, median (25th and 75th) | 11.62 (5.68, 17.00) | 12.48 (7.40, 15.26) | 9.95 (5.37, 17.04) | 0.528 |
| N×PLT/L, median (25th and 75th) | 2020.94 (766.86, 3239.21) | 2137.01 (993.25, 3227.79) | 1570.55 (582.52, 3384.31) | 0.44 |
| PLT/L, median (25th and 75th) | 22.84 (9.46, 36.48) | 23.85 (12.44, 36.06) | 18.32 (7.05, 40.23) | 0.471 |
| CRP/L, median (25th and 75th) | 23.98 (3.61, 79.09) | 14.09 (2.59, 74.64) | 31.91 (4.43, 85.24) | 0.278 |
| CRP/ALB, median (25th and 75th) | 0.73 (0.20, 2.43) | 0.33 (0.08, 2.21) | 0.85 (0.27, 2.46) | 0.181 |
| Arterial blood gas |  |  |  |  |
| PaO2, median (25th and 75th), mmHg | 83.50 (59.10, 158.50) | 68.25 (51.78, 133.25) | 96.70 (62.10, 213.00) | 0.110 |
| PaCO2, median (25th and 75th), mmHg | 31.10 (21.55, 39.65) | 29.40 (21.40, 37.20) | 31.60 (20.95, 40.95) | 0.387 |
| Lac, median (25th and 75th), mmol/L | 9.15 (6.10, 13.40) | 8.27 (4.03, 10.83) | 10.80 (7.18, 14.81) | 0.018 |
| pH, median (25th and 75th) | 7.24 (7.16, 7.35) | 7.28 (7.20, 7.36) | 7.20 (7.08, 7.34) | 0.018 |
| BE, median (25th and 75th), mmol/L | -13.40 (-19.80, -9.04) | -11.00 (-16.18, -7.95) | -14.90 (-21.73, -10.15) | 0.022 |
| HCO3-, median (25th and 75th), mmol/L | 15.10 (11.35, 18.00) | 15.80 (12.93, 18.80) | 14.40 (10.50, 17.20) | 0.139 |
| K+, median (25th and 75th), mmol/L | 4.15 (3.79, 4.81) | 4.10 (3.70, 4.48) | 4.47 (3.89, 5.00) | 0.159 |
| Na+, median (25th and 75th), mmol/L | 1.07 (1.00, 1.14) | 1.07 (0.99, 1.12) | 1.07 (1.02, 1.15) | 0.519 |
| Ca2+, median (25th and 75th), mmol/L | 140.00 (136.68, 142.30) | 140.00 (135.50, 143.00) | 140.00 (136.80, 142.00) | 0.680 |
| Cl-, median (25th and 75th), mmol/L | 107.00 (102.00, 109.75) | 107.00 (103.50, 110.23) | 107.00 (101.75, 109.25) | 0.840 |
| Coagulation profile |  |  |  |  |
| PT, median (25th and 75th), s | 14.05 (12.00, 16.58) | 13.60 (12.03, 16.63) | 14.85 (11.93, 16.78) | 0.652 |
| INR, median (25th and 75th) | 1.28 (1.10, 1.51) | 1.25 (1.09, 1.50) | 1.35 (1.09, 1.53) | 0.616 |
| FIB, median (25th and 75th), g/L | 2.94 (2.15, 3.89) | 2.82 (2.11, 3.81) | 3.27 (2.20, 4.48) | 0.423 |
| APTT, median (25th and 75th), s | 34.00 (30.60, 68.28) | 32.60 (30.53, 54.38) | 35.65 (30.68, 71.83) | 0.386 |
| TT, median (25th and 75th), s | 15.55 (13.40, 36.80) | 15.65 (13.53, 25.28) | 15.30 (13.10, 103.55) | 0.740 |
| D-D, median (25th and 75th), mg/L FEU | 2.24 (0.92, 6.86) | 2.64 (0.76, 6.02) | 2.14 (1.13, 10.15) | 0.356 |
| FDP, median (25th and 75th), mg/L | 5.94 (2.15, 14.87) | 8.36 (2.08, 13.71) | 5.32 (2.58, 17.22) | 0.630 |
| **Outcomes** |  |  |  |  |
| ECMO-related complications |  |  |  |  |
| Intracranial haemorrhage, n (%) | 2 (2.6%) | 0 (0.0%) | 2 (4.8%) | 0.497 |
| Gastrointestinal bleeding, n (%) | 8 (10.3%) | 0 (0.0%) | 8 (19.0%) | 0.017 |
| AKI, n (%) | 36 (46.2%) | 11 (30.6%) | 25 (59.5%) | 0.011 |
| Duration of ECMO support, median (25th and 75th), h | 117.5 (72.5, 176.5) | 136.3 (81.6, 192.5) | 112.8 (49.3, 168.5) | 0.159 |
| Total hospital stay, median (25th and 75th), days | 11 (5, 18) | 18 (9, 24) | 6 (2, 12) | <0.001 |
| ICU stay, median (25th and 75th), days | 9 (4, 16) | 14 (8, 22) | 6 (2, 12) | <0.001 |

BMI, body mass index; PCI, percutaneous coronary intervention; SOFA, SOFA, Sequential Organ Failure Assessment; SAVE, Survival After Veno-arterial ECMO; AMI, acute myocardial infarction; CAG, coronary angiography; LM, left main (coronary artery); LAD, left anterior descending (coronary artery); LCX, left circumflex (coronary artery); RCA, right coronary artery; CTO, chronic total occlusion; TIMI, thrombolysis in myocardial infarction (flow grade); EF, ejection fraction; FS, fractional shortening; SV, stroke volume; LVEDV, left ventricular end-diastolic volume; LVESV, left ventricular end-systolic volume; SI, stroke index; CO, cardiac output; CI, cardiac index; ALT, alanine aminotransferase; AST, aspartate aminotransferase; TBIL, total bilirubin; DBIL, direct bilirubin; IBIL, indirect bilirubin; Urea, blood urea nitrogen; Cr, creatinine; TP, total protein; ALB, albumin; GLB, globulin; ALP, alkaline phosphatase; GGT, gamma-glutamyl transferase; Glu, glucose; TC, total cholesterol; TG, triglyceride; HDL-C, high-density lipoprotein cholesterol; LDL-C, low-density lipoprotein cholesterol; LDH, lactate dehydrogenase; TyG index, triglyceride-glucose index; PCT, procalcitonin; CRP, c-reactive protein; IL-6, interleukin-6; WBC, white blood cell count; RBC, red blood cell count; HB, hemoglobin; PLT, platelet count; NEUT%, neutrophil percentage; NEUT, neutrophil count; LYM%, lymphocyte percentage; LYM, lymphocyte count; MONO%, monocyte percentage; MONO, monocyte count; EO%, eosinophil percentage; EO, eosinophil count; BA%, basophil percentage; BA, basophil count; L/N, lymphocyte-to-neutrophil ratio; L/M, lymphocyte-to-monocyte ratio; N/L, neutrophil-to-lymphocyte ratio; N×PLT/L, neutrophil–platelet–lymphocyte index; PLT/L, platelet-to-lymphocyte ratio; CRP/L, c-reactive protein-to-lymphocyte ratio; CRP/ALB, c-reactive protein-to-albumin ratio; PaO₂, partial pressure of oxygen; PaCO₂, partial pressure of carbon dioxide; Lac, lactate; pH, potential of hydrogen; BE, base excess; HCO₃⁻, bicarbonate; K⁺, potassium; Na⁺, sodium; Ca²⁺, calcium; Cl⁻, chloride; PT, prothrombin time; INR, international normalized ratio; FIB, fibrinogen; APTT, activated partial thromboplastin time; TT, thrombin time; D-D, d-dimer; FDP, fibrin degradation products; ECMO, extracorporeal membrane oxygenation; AKI, acute kidney injury; ICU, intensive care unit.

**Supplementary Table 13.** Cardiac biomarkers and dynamic changes in SCAI D.

|  | **Total (n=78)** | **Survivor（n=36）** | **Non-survivor (n=42)** | ***P*** |
| --- | --- | --- | --- | --- |
| **Hospital admission** |  |  |  |  |
| TnI, median (25th and 75th), ng/mL | 8.00 (1.20, 25.00) | 10.35 (1.63, 25.00) | 6.70 (1.14, 24.25) | 0.330 |
| CK-MB, median (25th and 75th), ng/mL | 104.00 (30.50, 473.50) | 104.00 (41.00, 500.00) | 114.00 (23.50, 446.25) | 0.401 |
| Myo, median (25th and 75th), ng/mL | 900.00 (593.00, 900.00) | 900.00 (859.00, 900.00) | 900.00 (368.00, 900.00) | 0.134 |
| NT-proBNP, median (25th and 75th), pg/mL | 5330.00 (1580.00, 10800.00) | 4660.00 (696.00, 11000.00) | 5970.00 (1855.00, 10375.00) | 0.623 |
| D-dimer, median (25th and 75th), ng/mL | 2500.00 (852.75, 7310.00) | 1710.00 (742.00, 4540.00) | 3730.00 (1370.00, 8805.00) | 0.077 |
| **6 h postoperatively** |  |  |  |  |
| TnI, median (25th and 75th), ng/mL | 25.00 (8.60, 25.00) | 25.00 (21.25, 25.00) | 14.00 (4.15, 25.00) | 0.007 |
| CK-MB, median (25th and 75th), ng/mL | 412.00 (47.00, 500.00) | 500.00 (148.75, 500.00) | 271.00 (31.00, 500.00) | 0.052 |
| Myo, median (25th and 75th), ng/mL | 900.00 (530.00, 900.00) | 900.00 (539.00, 900.00) | 900.00 (360.00, 900.00) | 0.369 |
| **24 h postoperatively** |  |  |  |  |
| TnI, median (25th and 75th), ng/mL | 18.00 (6.50, 25.00) | 24.00 (7.70, 25.00) | 12.50 (3.53, 25.00) | 0.094 |
| CK-MB, median (25th and 75th), ng/mL | 179.00 (47.00, 396.00) | 195.50 (57.00, 355.75) | 169.00 (39.00, 482.00) | 0.897 |
| Myo, median (25th and 75th), ng/mL | 823.50 (302.25, 900.00) | 544.00 (279.00, 900.00) | 900.00 (588.00, 900.00) | 0.023 |
| NT-proBNP, median (25th and 75th), pg/mL | 5580.00 (2715.00, 9477.50) | 5100.00 (2330.00, 7747.50) | 5690.00 (4015.00, 10742.50) | 0.317 |
| D-dimer,, median (25th and 75th), ng/mL | 1800.00 (1240.00, 5820.00) | 1770.00 (926.50, 3757.50) | 1930.00 (1265.00, 11780.00) | 0.467 |
| ΔTnI (6 - 0 h), median (25th and 75th), mmol/L | 1.10 (0.00, 15.30) | 5.35 (0.00, 23.43) | 1.00 (0.00, 9.55) | 0.179 |
| ΔCK-MB (6 h - 0 h), median (25th and 75th), mmol/L | 7.00 (0.00, 229.25) | 9.00 (-3.00, 374.00) | 4.50 (0.00, 102.00) | 0.391 |
| ΔMyo (6 - 0 h), median (25th and 75th), mmol/L | 0.00 (-36.00, 4.50) | 0.00 (-213.00, 0.00) | 0.00 (-13.50, 53.00) | 0.349 |
| ΔTnI (24 - 6 h), median (25th and 75th), mmol/L | 0.00 (-4.75, 0.00) | 0.00 (-5.95, 0.00) | 0.00 (-4.20, 0.00) | 0.199 |
| ΔCK-MB (24 - 6 h), median (25th and 75th), mmol/L | -74.00 (-192.75, 0.00) | -132.00 (-279.50, -13.00) | -10.00 (-134.00, 0.00) | 0.010 |
| ΔMyo (24 - 6 h), median (25th and 75th), mmol/L | 0.00 (-263.00, 0.00) | -154.00 (-369.00, 0.00) | 0.00 (-26.00, 17.00) | 0.002 |
| ΔTnI (24 - 0 h), median (25th and 75th), mmol/L | 1.36 (0.00, 13.00) | 2.60 (0.00, 20.04) | 1.00 (-0.26, 8.01) | 0.430 |
| ΔCK-MB (24 - 0 h), median (25th and 75th), mmol/L | -0.50 (-133.50, 99.75) | -16.50 (-179.00, 97.00) | 0.00 (-119.50, 110.25) | 0.370 |
| ΔMyo (24 - 0 h), median (25th and 75th), mmol/L | -4.00 (-371.00, 0.00) | -176.00 (-529.25, 0.00) | 0.00 (-24.00, 24.00) | 0.001 |
| ΔNT-proBNP (24 - 0 h), median (25th and 75th), mmol/L | 920.00 (-2560.00, 3812.50) | 969.00 (-3212.50, 2844.75) | 920.00 (-2510.00, 4547.50) | 0.743 |
| ΔD-dimer, (24 - 0 h), median (25th and 75th), mmol/L | 310.00 (-3737.5, 2156.75) | 206.00 (-2880.00, 1299.00) | 414.00 (-4760.00, 3375.00) | 0.878 |

TnI, troponin I; CK-MB, creatine kinase–MB isoenzyme; Myo, myoglobin; NT-proBNP, n-terminal pro–b-type natriuretic peptide; Lac, lactate; ΔTnI (6 - 0 h), the difference between the troponin I level at 6 h post-pci and that on admission (0 h); ΔCK-MB (6 - 0 h), the difference between the creatine kinase–mb isoenzyme level at 6 h post-pci and that on admission; ΔMyo (6 - 0 h), the difference between the myoglobin level at 6 h post-pci and that on admission; ΔTnI (24 - 6 h), the difference between the troponin I level at 24 h and 6 h post-pci; ΔCK-MB (24 - 6 h), the difference between the creatine kinase–mb isoenzyme level at 24 h and 6 h post-pci; ΔMyo (24 - 6 h), the difference between the myoglobin level at 24 h and 6 h post-pci; ΔTnI (24 - 0 h), the difference between the troponin I level at 24 h post-pci and that on admission; ΔCK-MB (24 - 0 h), the difference between the creatine kinase–mb isoenzyme level at 24 h post-pci and that on admission; ΔMyo (24 - 0 h), the difference between the myoglobin level at 24 h post-pci and that on admission; ΔNT-proBNP (24 - 0 h), the difference between the n-terminal pro–b-type natriuretic peptide level at 24 h post-pci and that on admission; ΔD-Dimer (24 - 0 h), the difference between the d-dimer level at 24 h post-pci and that on admission.

**Supplementary Table 14.** Lactate levels and dynamic changes in SCAI D.

|  | **Total (n=78)** | **Survivor（n=36）** | **Non-survivor (n=42)** | ***P*** |
| --- | --- | --- | --- | --- |
| Lac 0 h, median (25th and 75th), mmol/L | 9.15 (6.10, 13.40) | 8.27 (4.03, 10.83) | 10.80 (7.18, 14.81) | 0.018 |
| Lac 6 h, median (25th and 75th), mmol/L | 4.60 (2.68, 7.13) | 3.50 (2.35, 5.30) | 5.40 (3.70, 10.68) | 0.013 |
| Lac 2 4h, median (25th and 75th), mmol/L | 2.20 (1.40, 3.40) | 1.70 (1.30, 2.43) | 2.60 (1.70, 4.50) | 0.006 |
| ΔLac (6 - 0 h), median (25th and 75th), mmol/L | -4.95 (-8.42, -1.58) | -5.45 (-8.49, -1.11) | -4.75 (-8.05, -1.90) | 0.760 |
| ΔLac (24 - 0 h), median (25th and 75th), mmol/L | -7.72 (-11.60, -3.00) | -7.65 (-10.07, -2.86) | -8.27 (-13.75, -3.70) | 0.227 |
| ΔLac (24 - 6 h), median (25th and 75th), mmol/L | -0.20 (-3.20, 0.00) | -0.50 (-2.30, 0.00) | -0.20 (-3.60, 0.00) | 0.860 |

Lac 0 h, lactate level on admission; Lac 6 h, lactate level at 6 h post-PCI; Lac 24 h, lactate level at 24 h post-PCI; ΔLac (6 - 0 h), the difference between lactate levels at 6 h post-PCI and on admission; ΔLac (24 - 0 h), the difference between lactate levels at 24 h post-PCI and on admission; ΔLac (24 - 6 h), the difference between lactate levels at 24 h and 6 h post-PCI.

**Supplementary Table 15.** Multivariate analysis in SCAI D.

|  | **B** | **P** | **OR** | **95%CI** |
| --- | --- | --- | --- | --- |
| ΔCK-MB (24 - 6 h) | 0.003 | 0.186 | 1.003 | 0.998−1.008 |
| ΔMyo (24 -6 h) | 0.001 | 0.641 | 1.001 | 0.998−1.003 |
| ΔMyo (24 -0 h) | 0.002 | 0.137 | 1.002 | 0.999−1.004 |

ΔCK-MB (24 - 6 h), the difference between the creatine kinase–MB isoenzyme level at 24 h and 6 h post-PCI; ΔMyo (24 - 6 h), the difference between the myoglobin level at 24 h and 6 h post-PCI; ΔMyo (24 - 0 h), the difference between the myoglobin level at 24 h post-PCI and that on admission; OR, odds ratio; 95% CI, confidence interval.


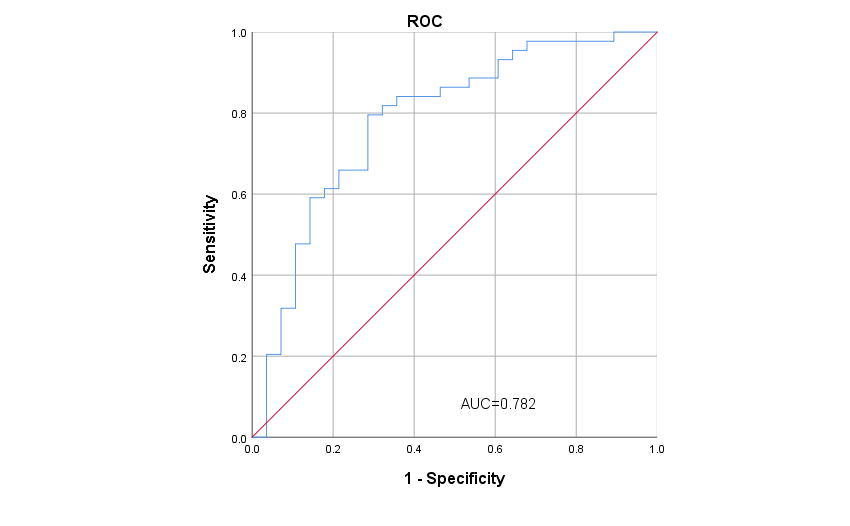


**Supplementary Fig. 1.** Receiver operating characteristic (ROC) curves of the prediction model and other indicators for the period 2020-2022.


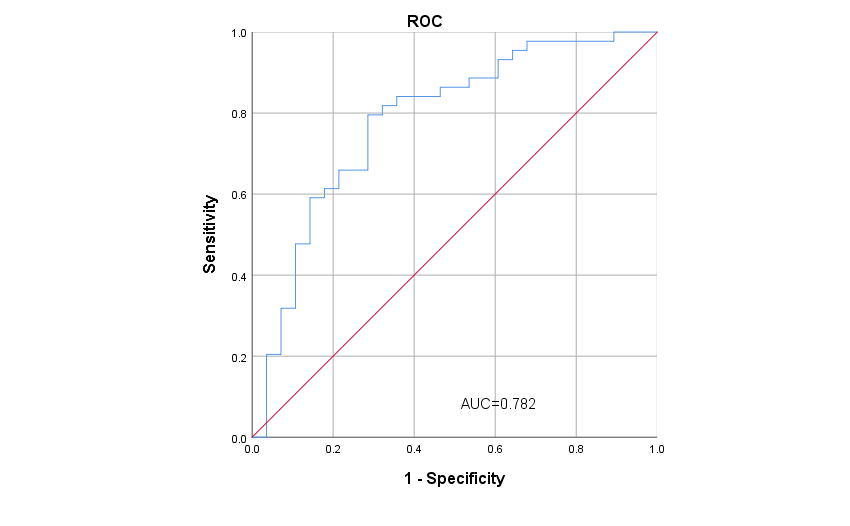


**Supplementary Fig. 2.** Receiver operating characteristic (ROC) curves of the prediction model and other indicators for the period 2023.
